# Real-time assessment of hypnotic depth, using an EEG-based brain-computer interface: a preliminary study

**DOI:** 10.1186/s13104-023-06553-2

**Published:** 2023-10-24

**Authors:** Nikita V. Obukhov, Peter L.N. Naish, Irina E. Solnyshkina, Tatiana G. Siourdaki, Ilya A. Martynov

**Affiliations:** 1 Research Department, The Association of Experts in the Field of Clinical Hypnosis, 40, Kamennoostrovsky Ave., 410, Saint Petersburg, 197022 Russian Federation; 2 Department of Psychotherapy, Academician I.P. Pavlov First St. Petersburg State Medical University, 6-8, L. Tolstoy str, Saint Petersburg, 197022 Russian Federation; 3 Department of Psychology, The Open University, Walton Hall, Milton Keynes, MK7 6AA UK

**Keywords:** Hypnosis, Hypnotic depth assessment, Supervised machine learning, Brain-computer interface, Awareness, Self-awareness

## Abstract

**Objective:**

Hypnosis can be an effective treatment for many conditions, and there have been attempts to develop instrumental approaches to continuously monitor hypnotic state level (“depth”). However, there is no method that addresses the individual variability of electrophysiological hypnotic correlates. We explore the possibility of using an EEG-based passive brain-computer interface (pBCI) for real-time, individualised estimation of the hypnosis deepening process.

**Results:**

The wakefulness and deep hypnosis intervals were manually defined and labelled in 27 electroencephalographic (EEG) recordings obtained from eight outpatients after hypnosis sessions. Spectral analysis showed that EEG correlates of deep hypnosis were relatively stable in each patient throughout the treatment but varied between patients. Data from each first session was used to train classification models to continuously assess deep hypnosis probability in subsequent sessions. Models trained using four frequency bands (1.5–45, 1.5–8, 1.5–14, and 4–15 Hz) showed accuracy mostly exceeding 85% in a 10-fold cross-validation. Real-time classification accuracy was also acceptable, so at least one of the four bands yielded results exceeding 74% in any session. The best results averaged across all sessions were obtained using 1.5–14 and 4–15 Hz, with an accuracy of 82%. The revealed issues are also discussed.

**Supplementary Information:**

The online version contains supplementary material available at 10.1186/s13104-023-06553-2.

## Introduction

Hypnosis can be an effective treatment for numerous disorders [1–3]. The hypnotic response may depend on factors such as hypnotisability, the hypnotic state, expectations [4–6], motivation, the therapeutic relationship, etc. [7]. Hypnotisability is a personal trait that greatly affects treatment outcomes [8–13]. Nevertheless, hypnosis is currently defined as a state of consciousness [14], and this study is focused solely on the hypnotic state. Hypnotic “depth” has been a contentious issue, but it is still used in contemporary research to describe the hypnotic state quantitatively [5, 15–22], alongside the related concepts of “deep hypnosis” [6, 18, 19, 23–31] and “deepening” [16, 32–39]. Multiple studies indicate that sufficient depth is beneficial in some cases, e.g., in non-pharmacological analgesia [24, 25, 40], general hypnotic anaesthesia [26, 30, 33], etc. Greater depth could result in subjects’ feeling more influenced by hypnotic procedures, leading to better compliance [41, 42]. Depth self-ratings can correlate with hypnotisability scores [19].

Probably, a hypnotic state gradually evolves during a session and tends to fluctuate [43]. The possible usefulness of a more accurate estimation of subtle depth alterations, unable to be seen visually, led researchers to the idea of a “hypnometer” [40], a device for real-time hypnotic depth measures to help a practitioner decide whether to continue with deepening or to begin therapeutic suggestions. Heart rate variability (HRV) [40] and an EEG-based parameter, the bispectral index (BIS), were considered the bases for such measures. BIS is a promising method [36, 44]; however, its calculation algorithm is designed primarily for pharmacological anaesthesia rather than hypnotherapy. Moreover, although hypnosis is characterised by some common EEG correlates [18, 31, 36, 44–57], differences between subjects are also observed. Perhaps an approach that addresses individual variability could have benefits.

Passive brain-computer interfaces (pBCI) are used to assess mental states such as fatigue, concentration, etc. [58, 59]. We hypothesise that machine learning might be used to recognise and continuously quantify EEG correlates of hypnosis specific to a person. We aim to explore this possibility by designing a prototype system using an EEG-based pBCI to real-time monitor hypnosis deepening and conducting its initial feasibility test.

## Materials and methods

### Participants

Nine outpatients (six women, mean age: 38.33 ± 10.61 years) underwent up to seven hypnosis sessions. Inclusion criteria: age 18–65 years; consent for participation. Patients had previously reported experiencing deep hypnosis, described as a lack of self-awareness, external awareness, and memories for the deepest period of a session. Thus, probably all included participants could be classified as “dissociators” [60–62], “amnesia-prone” [63], or “dissociative” subtype individuals [64]. This is common, yet not the only type to experience hypnosis [4, 5, 15, 65]. Assumed neural correlates of such phenomena were shown in several studies [29, 66]. Exclusion criteria: cognitive decline, epilepsy, psychosis, and no episode of feeling deeply hypnotised during the first session. See “Supplements A(1)” for the participants’ summary.

### Hardware and software equipment

The BCI system included equipment for synchronised EEG and video recording and also software: WinEEG 2.130.101 [67], EEGLab 2019.0 [68], and OpenVibe 2.2.0 [69]. See “Supplements A(2)” for details.

### A brief description of a hypnotic session

In each patient, after installing the electrodes and equipment for EEG and video recording, a baseline EEG was recorded for 3–5 min with eyes closed. Hypnosis was then induced and deepened by the counting method. After therapeutic suggestions, a patient was awakened. Feedback was then collected.

### The principle of the proposed approach

We used the passive type of BCI [58, 59, 70] and supervised learning [71, 72]. A recording from the first session with each patient was used as a calibration file to train a classifier. We first manually identified and then labelled the EEG intervals corresponding to two opposite states of an implied neurophysiological continuum: wakefulness (which matched the baseline registration periods) and the deepest states. The timing of the deepest states was identified with two criteria that had to be present simultaneously:

- The physical signs recommended to verify sufficient depth [23, 24, 26]: substantial changes in breathing, relaxation of facial muscles, etc.
- The patients’ post-session feedback on the hypnotherapist’s counting range during which they felt most deeply hypnotised (see “Supplements A(3)” for this procedure’s details).

The file with two sets of labels was then used to train a classifier to continuously recognise (“predict”) these two opposite states in subsequent sessions with interdependent probability. Assuming that deepening is a continuous transition from wakefulness to the deepest hypnotic state, we hypothesise that the continual real-time measurement of the probability of a deep hypnosis during a session could tentatively, to some extent, operate as a quantitative reflection of the deepening process.

### Analysis and processing of obtained recordings

The collected data were processed in four ways.

- Using WinEEG to compare the averaged power spectra of the identified deep hypnosis and the waking periods (5–10 min each) in each EEG, we obtained an overview of each patient’s assumed deep hypnosis patterns (which rhythms at which locations tend to alter while deeply hypnotised). We also assessed their putative stability throughout the treatment, qualitatively comparing them over different patient sessions. This analysis was conducted in parallel with all the others.
- During supervised learning, we trained prediction models, and those derived from the first sessions were then used in the following sessions for real-time classification. The Common Spatial Pattern (CSP) [73–76] method was employed for signal spatial filtering, and Linear Discriminant Analysis (LDA) was a classification method. We tested four frequency bands: 1.5–45 Hz; 1.5–8 Hz; 1.5–14 Hz; and 4–15 Hz, to determine which could yield the models with the most classification accuracy, as assessed by the 10-fold cross-validation test. To get the cross-validation results for all sessions, the training procedure should be performed in each session as we did in the first (calibration) one. Thus, each second and subsequent session produced the “auxiliary” models. See “Supplements A(3)” for details.
- Using models derived from the first sessions for real-time state predictions in subsequent sessions yielded a Probability Value parameter, varying between 0 and 1, displayed as a moving curve, which informed the hypnotherapist of the probability of a deep hypnotic state occurring. We called this curve the Predictive curve. “Supplements A(4)” describe details.
- Each second and subsequent session was labelled by a specialist not privy to their predictive outcomes, and a percentage of correctly predicted states for different epochs was calculated to additionally test the accuracy [77]. The above-mentioned “auxiliary” models from these sessions were applied to classify the same data on which these models had been trained to plot a curve that reflected depth dynamics in a session most accurately—the Native curve. The Native and Predictive curves of the same sessions were then visually compared. For details, see “Supplements A(5)”.

### Results and their discussion

Patient T reported no periods of deep hypnosis in the first session and was excluded from the analysis. Due to the artefacts, the recording from session #5 of Patient E was also excluded. Thus, the total number of EEGs from the remaining eight participants was 27.

### Results of the qualitative assessment of the estimated patterns of deep hypnosis in patients and their stability throughout the treatment

**Fig. 1:**
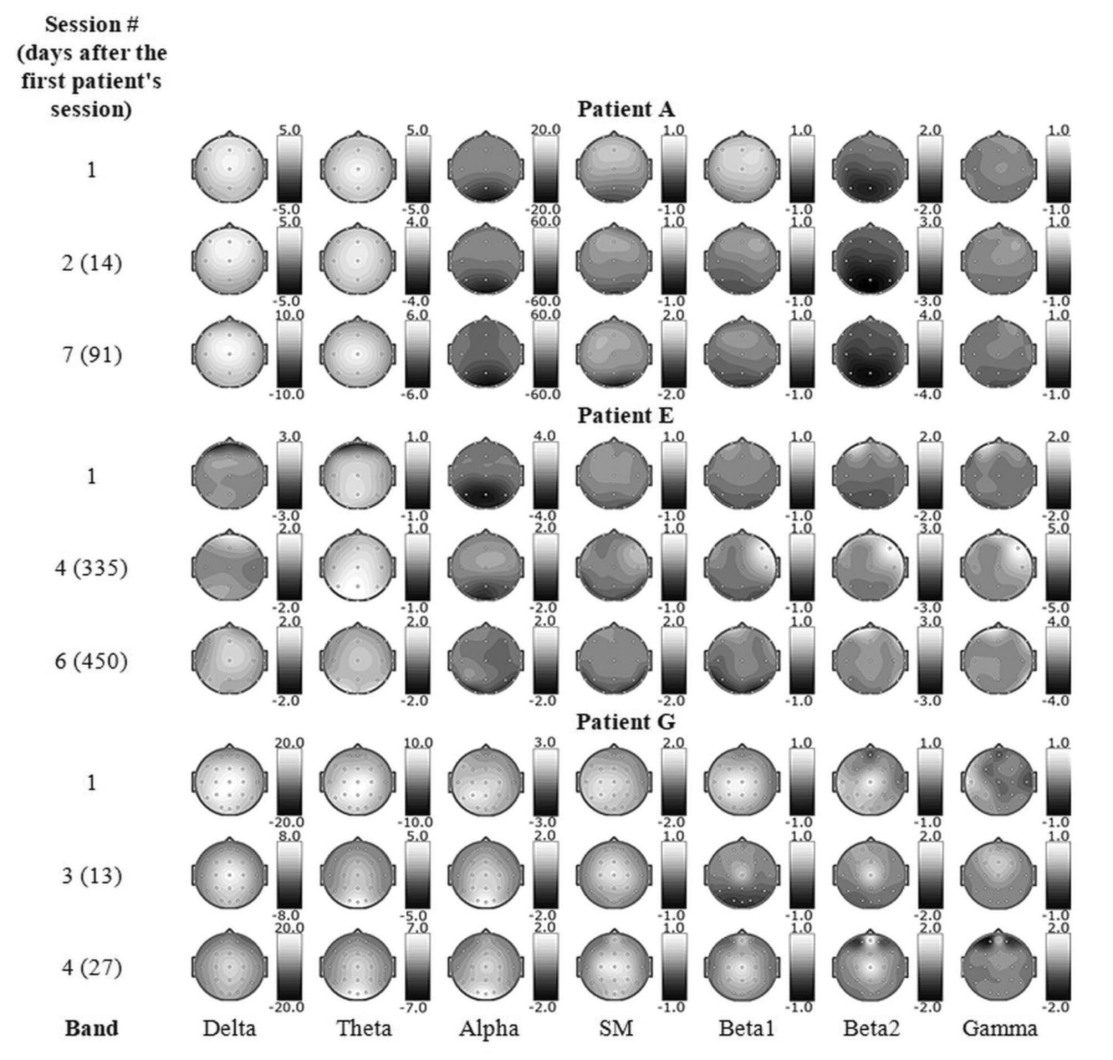
Comparison of power spectra between periods of deep hypnosis and wakefulness This is an example of topographic maps displaying the differences in the averaged power spectra between deep hypnosis and the waking periods of EEG (“deep hypnosis” minus “wakefulness”) for three patients. Sessions for display are arbitrarily selected. Thus, the maps demonstrate the alterations in the power of different rhythms for different localizations while achieving deep hypnosis. Power changes are displayed in colour according to the graduation of a nearby colour scale (in uV²). The bands used are: Delta (1.5-4 Hz), Theta (4–8 Hz), Alpha (8–12 Hz), Sensory-motor or Low beta (12–15 Hz), Beta1 (15–18 Hz), Beta2 (18–25 Hz) and Gamma (25–45 Hz)

Figure 1 demonstrates examples of topographic maps representing these results for three patients.

As seen, the electrophysiological changes tend to be generally similar in different sessions of a particular patient, which suggests that a classifier would correctly recognise the target patterns in the following sessions. Correlates common to the patients are seen, e.g., a slow-wave activity increase, which is consistent with the literature [18, 31, 45–57]. Several differences between individuals are also shown; therefore, an individualised approach to quantifying hypnosis appeared to be preferable. These points suggest that machine learning may potentially apply to hypnosis. However, the qualitative analysis is approximate, and further statistical assessment may be useful.

“Supplements B” contain all the maps for all 27 sessions for all patients and the details on electrophysiological changes revealed.

### Results of the 10-fold cross-validation test of classification accuracy

Table 1(A) shows that the accuracy exceeded 85% in the majority of cases. Results of 100% were possibly due to the optimistic calculation outcomes of the software [78] or the over-fitting phenomenon [79], which are undesirable and should be addressed further.

**Table 1:** Deep hypnosis and wakefulness classification accuracy according to the band used to train the models

Patient	Session	Accuracy for the corresponding band, %			
		1.5–45 Hz	1.5-8 Hz	1.5–14 Hz	4–15 Hz
(A) The 10-fold cross-validation test results, M ± SD					
A	1	96 ± 8	86 ± 18	98 ± 6	94 ± 9.17
	2	93.5 ± 10.01	95.5 ± 9.07	86.5 ± 14.33	72.5 ± 26.48
	3	88 ± 18.33	72 ± 31.24	86 ± 25.38	86 ± 25.38
	4	95 ± 15	83 ± 15.84	95 ± 10	95 ± 10
	5	94 ± 9.17	89.67 ± 17.16	92.33 ± 13	96.33 ± 7.37
	6	98 ± 6	87 ± 15.52	92 ± 13.27	96 ± 8
	7	84.17 ± 21.87	93.33 ± 13.33	83.33 ± 22.36	86.67 ± 16.33
	M ± SD	92.67 ± 4.86	86.64 ± 7.75	90.45 ± 5.32	89.5 ± 8.65
E	1	97.5 ± 7.5	82 ± 15.68	88.5 ± 16.13	95 ± 10
	2	100 ± 0	90.5 ± 11.72	93 ± 15.52	86.5 ± 19.11
	3	100 ± 0	91 ± 11.14	100 ± 0	100 ± 0
	4	95 ± 10	93 ± 15.52	91 ± 15.78	92 ± 24
	6*	100 ± 0	96 ± 8	94 ± 18	96 ± 8
	M ± SD	98.5 ± 2.24	90.5 ± 5.22	93.3 ± 4.3	93.9 ± 5.03
G	1	94.33 ± 8.7	96 ± 8	98.33 ± 5	96 ± 12
	2	98.33 ± 5	92.33 ± 18.14	94.67 ± 11.08	93 ± 15.52
	3	100 ± 0	100 ± 0	100 ± 0	96.33 ± 7.37
	4	98 ± 6	98 ± 6	98 ± 6	98 ± 6
	M ± SD	97.67 ± 2.39	96.58 ± 3.27	97.75 ± 2.23	95.83 ± 2.08
S	1	100 ± 0	100 ± 0	97.5 ± 7.5	95.5 ± 9.07
	2	97.5 ± 7.5	100 ± 0	100 ± 0	100 ± 0
	3	97.5 ± 7.5	97.5 ± 7.5	97.5 ± 7.5	97.5 ± 7.5
	M ± SD	98.33 ± 1.44	99.17 ± 1.44	98.33 ± 1.44	97.67 ± 2.25
O	1	100 ± 0	100 ± 0	97.5 ± 7.5	97.5 ± 7.5
	2	100 ± 0	100 ± 0	97.5 ± 7.5	87.5 ± 16.78
	M ± SD	100 ± 0	100 ± 0	97.5 ± 0	92.5 ± 7.07
N	1	96 ± 8	98 ± 6	94 ± 18	98 ± 6
	2	100 ± 0	100 ± 0	100 ± 0	100 ± 0
	M ± SD	98 ± 2.83	99 ± 1.41	97 ± 4.24	99 ± 1.41
V	1	91 ± 15.78	93.5 ± 10.01	98 ± 6	95.5 ± 9.07
	2	100 ± 0	98 ± 6	100 ± 0	100 ± 0
	M ± SD	95.5 ± 6.36	95.75 ± 3.18	99 ± 1.41	97.75 ± 3.18
C	1	100 ± 0	95 ± 15	81.67 ± 22.91	96.67 ± 10
	2	100 ± 0	77.5 ± 29.36	93.33 ± 20	93.33 ± 20
	M ± SD	100 ± 0	86.25 ± 12.37	87.5 ± 8.24	95 ± 2.36
(B) Results based on the data from the second (and subsequent) sessions					
A	2	74.31	52.08	74.65	66.32
	3	80.0	75.34	91.51	89.86
	4	99.48	64.83	87.66	88.98
	5	69.09	66.94	94.62	91.94
	6	90.85	69.82	90.24	87.8
	7	79.73	66.32	80.76	73.54
	M ± SD	82.24 ± 11.12	65.89 ± 7.72	86.57 ± 7.48	83.07 ± 10.52
E	2	54.75 (98.42**)	38.29	70.89	80.38
	3	54.18 (72.76***)	65.63	53.56	83.9
	4	71.82	69.7	81.52	84.55
	6*	70.06	44.19	59.01	84.01
	M ± SD	62.7 ± 9.54	54.45 ± 15.53	66.24 ± 12.49	83.21 ± 1.9
G	2	87.10	91.13	91.13	73.92
	3	88.98	97.31	90.05	81.72
	4	75.78	94.3	89.46	87.75
	M ± SD	83.95 ± 7.14	94.25 ± 3.09	90.21 ± 0.85	81.13 ± 6.93
S	2	53.03	93.64	95.76	53.64
	3	94.3	87.03	78.16	75.95
	M ± SD	73.67 ± 29.18	90.34 ± 4.67	86.96 ± 12.45	64.8 ± 15.78
O	2	61.46	89.58	89.24	88.89
N	2	97.09	93.6	96.8	96.51
V	2	95.06	90.41	88.08	94.48
C	2	71.92	61.15	66.15	82.31
The average for all sessions, M ± SD		77.32 ± 14.89	74.28 ± 17.96	82.59 ± 12.53	82.44 ± 10.35

(A) The results of the 10-fold cross-validation test are shown as the average classification accuracy among the ten partitions of the marked file and the corresponding standard deviation

(B) This accuracy is calculated based on the data from the second and subsequent sessions as a percentage of coincidence between the states predicted by the model for different EEG epochs and the actual states related to these epochs

* Session 5 has been excluded from the analysis

**Value after a drift correction with a subtraction of 1.5 from the original feature vector (for details, see “Supplements D”)

*** Value after a drift correction with a subtraction of 2.5 from the original feature vector (for details, see “Supplements D”)

### Results of real-time visual testing of the method (in the second and subsequent sessions)

**Fig. 2:**
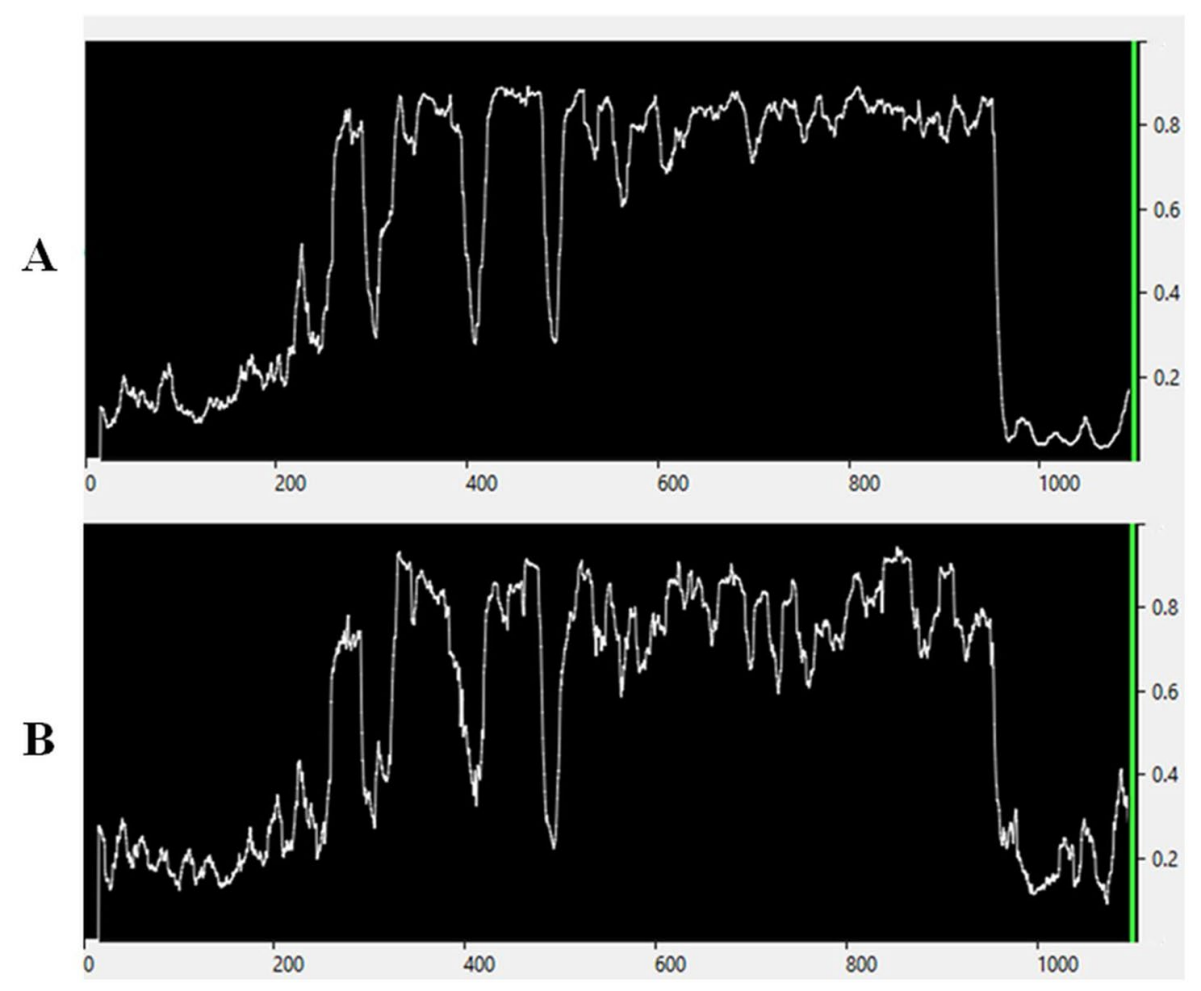
Examples of the Predictive (**A**) and Native (**B**) curves. The horizontal axis is a time scale (s), and the vertical axis is a Probability Value scale (from 0 to 1). (**A**) The Predictive curve graph was played back using data from the seventh session of Patient A. In this example, the classification model was trained using the 1.5–14 Hz range. The curve was smoothed by the Moving epoch average (Immediate) function. The number of 4-s epochs with an overlap of 0.5 s used for averaging was 50. This curve was obtained by feeding the real-time patient’s EEG during the seventh session to the model trained on data from the first session and represents the changing probability of a deep hypnotic state over the session. “Supplements C” contain a detailed case-related analysis of how it could potentially describe session dynamics. (**B**) The Native curve of the seventh session with Patient A. The band and the smoothing features are the same as in the Predictive curve. This curve was obtained after this (seventh) session by training a model (auxiliary) on data from the same session and then feeding this EEG recording to this model. Thus, this curve, which could only be constructed after the session was over, reflects the changes in the probability of deep hypnosis with very high accuracy

As an example of such results, Fig. 2(A) shows Patient A’s Predictive curve from his seventh session.

“Supplements C” contain the Predictive curves for each patient. In general, post-session patients’ reports revealed an approximate match between the time when a curve was consistently above 0.7 (approximately, depending on smoothing settings) and a period of unawareness. The high-amplitude wave-like motion was associated with alternating awareness, fractional memories, etc. Thus, this could potentially confirm instrumentally the literature reports of hypnotic depth variations during a session [43]. “Supplements C” contain a detailed analytical description using the individual case.

### Results of the classification accuracy evaluation based on the data from the second and subsequent sessions

Table 1(B) shows these results. As seen, the accuracy was high in many cases. Some poor results were due to either myographic artefacts or drift [80]. For a detailed analysis of the case-related findings, see “Supplements D”. In each session, at least one band yielded an accuracy exceeding 74%. Also, as seen, each patient had their “preferred” band for which the model was most accurate, e.g., for Patient A, it was 1.5–14 Hz; for Patients G and S, 1.5–8 Hz, etc., which might correspond with the literature observing variation in findings on electrophysiological correlates of hypnosis [31].

The accuracy averaged across all these sessions was highest when using bands 1.5–14 and 4–15 Hz. This is also in line with studies that reported the most changes in alpha and theta activity [18, 31, 36, 51, 54, 55].

### Results of a visual comparison of the Predictive and Native curves

Figure 2(B) shows the Native curve of Patient A’s seventh session. The configurations of curves (A) and (B) largely coincide, giving us additional confirmation that the model can reflect the real picture relatively accurately. “Supplements C” contain the pairs of the Predictive and Native curves for each patient for the second and following sessions.

This study extends the idea of a “hypnometer” [40] but focuses on direct monitoring of brain activity rather than peripheral measurements. BIS for this purpose is promising [36, 44]; however, we suggest that a new approach, which addresses individual correlates of neural activity, may have benefits. It could be used to optimise therapy by controlling depth more precisely when sufficient depth can be helpful [24–26, 30, 32, 33, 40–43, 81].

The designed system is a trial version only and requires further substantial improvements, using both the results and issues we revealed. However, we suppose it could initially demonstrate that pBCI applies to hypnosis.

### Limitations

- Small sample size, the heterogeneous number of sessions, and the artefacts in some recordings.
- Involvement only of those patients who described their deepest experiences as a lack of awareness and self-awareness. Although these phenomena are common for hypnotic experiences [4, 5, 15, 29, 65, 66], they are probably only characteristics of “dissociators” [60–62] or “amnesia-prone” individuals [63]. Perhaps the “fantasizers” [60–62] or “fantasy-prone” individuals [63] occurred outside of our focus, and further research should include this group.
- We did not measure the participants’ hypnotisability. Trait effects are considered to have a different basis [4–7, 9], and the research of the interaction of state and trait is a serious task that we believe deserves a separate investigation.
- A single rater was used to label each given recording.
- Using the measurement of the continuously changing deep hypnosis probability as a tentative reflection of the deepening process is a hypothetical idea in the early stages of testing. The qualitative analysis conducted could partly underpin the information from a Predictive curve, but it is not comprehensive. Further studies, utilising the classification of intermediate levels of hypnosis, are needed.
- The qualitative analysis of estimated hypnotic patterns is approximate, and further studies can incorporate statistical assessment to strengthen these findings as well as quantify differences in accuracies across subjects and bands and identify similarities between the Native and Predictive curves.
- Our system is a prototype only, and the signal processing techniques used are not comprehensive, being the initial option. Further research is necessary to test other feature extraction approaches and classification methods [74, 82].

### Electronic supplementary material

Below is the link to the electronic supplementary material.

Supplementary Material 1(“Supplements A”)

Supplementary Material 2 (“Supplements B”)

Supplementary Material 3 (“Supplements C”)

Supplementary Material 4 (“Supplements D”)

## Data Availability

All data generated or analysed during this study are included in this published article and its supplementary information files.

## List of abbreviations

EEG: Electroencephalography
BCI: Brain-computer Interface
pBCI: Passive Brain-computer Interface
HRV: Heart Rate Variability
BIS: Bispectral Index
CSP: Common Spatial Pattern
LDA: Linear Discriminant Analysis
