# Supplementary material for: Real-time assessment of hypnotic depth, using an EEG-based brain-computer interface: a preliminary study
Source: BMC Res Notes. 2023 Oct 24;16:288. doi: 10.1186/s13104-023-06553-2 (PMC10599062; doi:10.1186/s13104-023-06553-2)
Supplement: Supplementary file 1 — Supplementary Material 1(“Supplements A”) [file 13104_2023_6553_MOESM1_ESM.docx]

**Supplements A**

1. **Participants' data**

| **Patient's Code** | **Gender** | **Age, years** | **Diagnosis, ICD-10** | **Number of sessions** | **Medications, mg per day** |
| --- | --- | --- | --- | --- | --- |
| A | M | 34 | F41.1  Generalized anxiety disorder + F45.8  Other somatoform disorder | 7 | Tofisopam, 100 |
| E | F | 58 | F40.0  Agoraphobia | 6 | Escitalopram, 10 |
| G | F | 45 | F41.1  Generalized anxiety disorder | 4 | Sertraline, 50 |
| S | F | 49 | F40.01  Agoraphobia with panic disorder | 3 | Escitalopram, 10 |
| O | F | 33 | F32.1  Moderate depressive episode + F41.1  Generalized anxiety disorder | 2 | Fluoxetine, 40 |
| N | M | 35 | F41.1  Generalized anxiety disorder | 2 | Fluoxetine, 20 |
| V | M | 35 | F41.1  Generalized anxiety disorder | 2 | - |
| C | F | 22 | F32.1  Moderate depressive episode + F41.1  Generalized anxiety disorder | 2 | Sertraline, 100 |
| T | F | 34 | F41.1  Generalized anxiety disorder | 1 | - |

1. **Hardware and software equipment**

For EEG recording, we used a Mitsar-EEG-SmartBCI 21-channel neurointerface (CE medically certified (MDD 93/42/EEC), the hardware sampling rate was 2000 Hz filtered down to 250 Hz, and the frequency range was DC(0)–70 Hz) in combination with an elastic textile cap with fixing rings for point Ag/AgCl sintered electrodes located according to the international system 10–20 in positions: Fp1, Fpz, Fp2, F7, F3, Fz, F4, F8, T3, C3, Cz, C4, T4, T5, P3, Pz, P4, T6, O1, Oz, O2. The ground electrode was located in the AFz position, and the reference ones were on the earlobes (positions A1, A2). A monopolar montage regarding the ear electrodes A1 and A2 was used so that the left hemisphere leads are recorded in reference to A1, and the rights are recorded in reference to A2 (Fp1-A1, Fpz-A2, Fp2-A2, F7-A1, F3-A1, Fz-A1, F4-A2, F8-A2, T3-A1, C3-A1, Cz-A2, C4-A2, T4-A2, T5-A1, P3-A1, Pz-A1, P4-A2, T6-A2, O1-A1, Oz-A2, O2-A2). The impedance was maintained at a level below 5 kOhm. The video was recorded using a standard web camera.

1. **Offline classifier training and the 10-fold cross-validation test of classification accuracy**

Based on an EEG recording from the first patient's session, a classifier was trained to discriminate between two states: waking and deeply hypnotised, to be applied in subsequent sessions for the real-time prediction of the states. To provide this training, we first had to manually identify and then label the EEG intervals corresponding to these two opposite states of the inferred neurophysiological continuum. To define periods of deep hypnosis in a recording, two measures were employed. According to the recommendations on sufficient depth verification [1–3], the first of them picked times when the patients' facial muscles became "smooth", the lowering of the mandible was observed (occasionally accompanied by a slight mouth opening), significant changes in breathing frequency were detected, etc., all of which reflect the autonomic nervous system (ANS) alterations during hypnosis described in the literature [4–9]. The other was based on the post-session patients' reports of which periods they felt most deeply hypnotised during the session they had just completed. The patients were able to identify the range of numbers (from the hypnotherapist's counting) they were unaware of. They also reported a lack of self-awareness for that portion of the session and no memories of its content. As mentioned, these phenomenological markers were selected primarily because all our participants subjectively estimated them as the deepest hypnotic experience they had ever had. We don't claim that these phenomena are the only type of deep hypnosis experience, but merely that they were common to all patients in our study. We assume that some other phenomena might also potentially be used as markers of the deepest stage, depending on what the patient and the hypnotherapist decide to consider as such. Hypothetically, the alternatives could be the great vividness and strength of a suggested image [10], which is more specific for Barrett's "fantasizers" subtype [11–13], etc. Both types of criteria had to be present simultaneously (overlapped) in some period of a session to consider the period as corresponding to a deep state. Each session was video recorded synchronously with the EEG to accurately determine the timing of the physical signs of deep hypnosis.

For the training procedure, within each of the two types of periods in the calibration recording, we placed event labels: "W" (for wakefulness) and "D" (for the deep state), so that they could be used to specify the order of 20–25 short trial epochs for each state (the datasets were balanced). We attached the "D" marks to the moments of visual signs of sufficient depth *only within* intervals that patients reported as periods of deepest hypnotic experience." The "W" labels were placed arbitrarily along the phases of baseline EEG registration. Further, by passing this labelled file through a sequence of OpenVibe scenarios, we trained a prediction model to be used in the next sessions in real time. Generally, we had to determine three components for classification: a) the selected frequency band for band-pass filtering of the EEG signal, and depending on it, both b) coefficients of spatial filters, and c) the configuration file of the trained model containing relevant coefficients for the real-time classification process. It was necessary to define what frequency band could give us the models with the most predictive power. The wide band (1.5–45 Hz) was tested, and three narrower bands were also tested: 1.5–8 Hz, 1.5–14 Hz, and 4–15 Hz.

To increase the classification accuracy, we used spatial filtering of signals with the Common Spatial Pattern (CSP) method [14–17]. Scenario-I (see Figure S1 below) allowed us to calculate the spatial filter coefficients. To accomplish this, epochs of 4 s were allocated around each event label so that the first 0.5 s of the epoch preceded the label and the remaining 3.5 s followed it. The Temporal filter box used the band-pass filter by the Butterworth method, and the filter order was 5. The selected dimension in the CSP Spatial Filter Trainer box was 12.

**Figure S1.** Scenario-I in OpenVibe Designer calculates the spatial filter coefficients using the CSP algorithm. Each box contains a short functionality description on the left


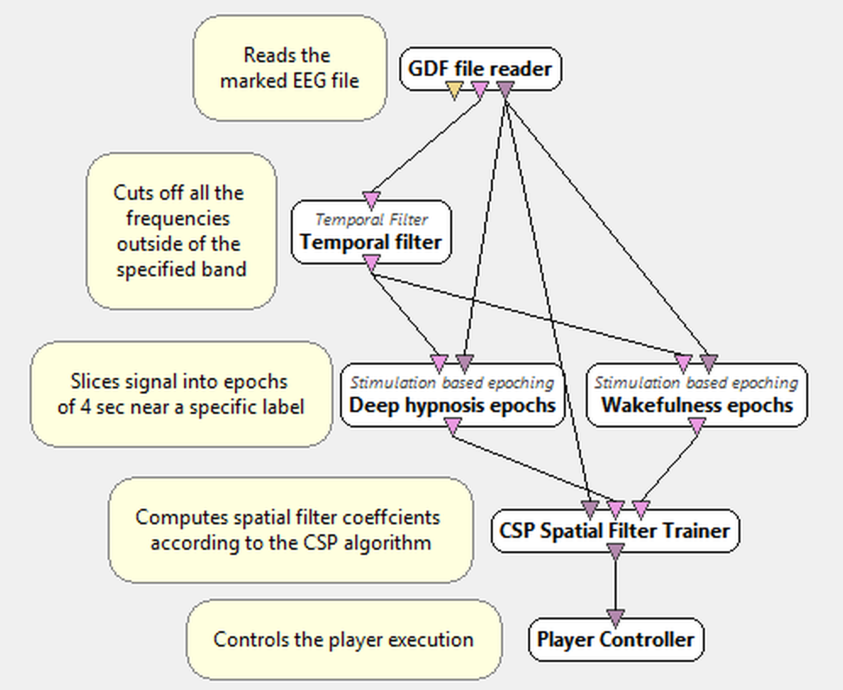


The obtained configuration files containing the spatial filter coefficients (component "b") were used in the following scenario (Scenario-II, see Figure S2 below), which is designed for classifier training. In Scenario-II, after frequency and spatial filtering, the signal was divided into two groups of epochs, as in the previous scenario. Further, the feature extraction was performed for each of these two groups: the logarithmic band power was computed as log (1+x), where "x" is the mean of the squares of the signal in each of the selected epochs. This value was subsequently converted into a feature vector, which then went to the Classifier Trainer box. Thus, the Classifier Trainer was receiving two types of feature vectors (corresponding to the states "W" and "D"), and after the end of the training, it produced a configuration file containing the relevant coefficients of the trained model (component "c") to be used during real-time classifications in the next scenario. Linear discriminant analysis (LDA) was applied as an algorithm to classify two types of feature vectors.

The usage of the proposed method in the therapeutic context assumes that EEG recordings of the first session will serve as a calibration file, but for research purposes, we labelled the recordings of each session and trained the models (using Scenario-I and -II) in each of them individually to calculate the classification accuracy for each given session. The Classifier Trainer box executed a 10-fold cross-validation test for four types of our models to estimate which frequency band provided the most accurate average results.

**Figure S2.** Scenario-II in OpenVibe Designer. Each box contains a short functionality description on the left, except those already described in Scenario-I


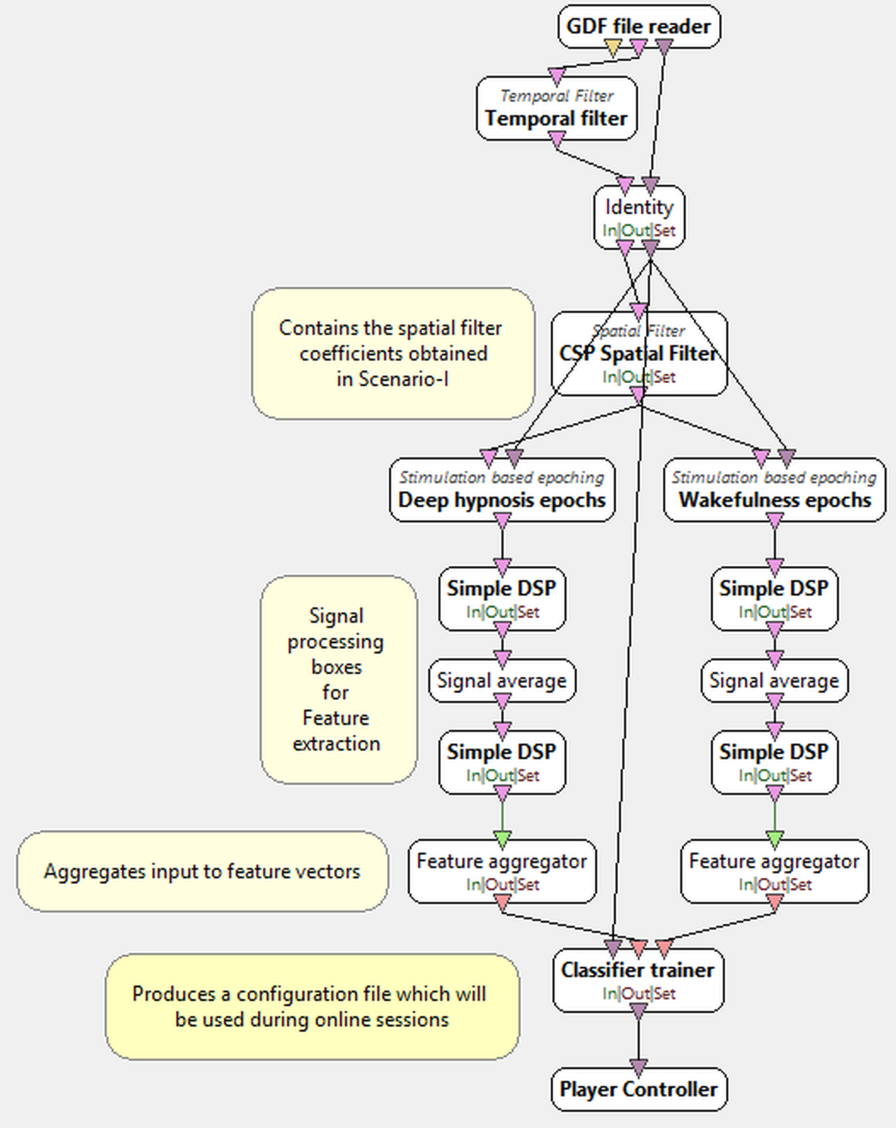


1. **Testing the trained models in real time**

All three obtained components ("a", "b," and "c") were applied in the second and following sessions to provide the real-time classification process. In Scenario-III (see Figure S3 below), the real-time EEG signal underwent band-pass and spatial filtering; epochs of 4 s of continuous filtered data were taken each 0.5 s using the Time based epoching box to be transformed into feature vectors like in Scenario-II. The feature vectors were then sent to the Classifier Processor box, which contained the configuration file of our trained model (component "c"). As a result, the system was able to predict in which of the two states and with what probability the patient was at any given moment, providing Probability Values as an output parameter. This output is a matrix of interdependent probabilities of matching current EEG patterns to one state and another. For visualisation convenience, we selected from the matrix only the probability of deep hypnosis and displayed this value fluctuating from 0 to 1 in real time using the Continuous Oscilloscope box. At that point, the closer to "1", the more probable the deepest stage and the less probable the wakefulness. Assuming that deepening is a continuous transition from wakefulness to the deepest hypnotic state, we hypothesise that the continual real-time measurement of the probability of a deep hypnosis during a session could tentatively, to some extent, operate as a quantitative reflection of the deepening process. We realise that this is largely just an assumption that must undergo several stages of verification. In this study, we try to make the first attempts. Thus, Scenario-III was built to provide a single integrative, individualised, continuously changing parameter displayed in the form of a curve during a session. We called this curve the Predictive curve. For the convenience of displaying data over a large time interval, to smooth the curve, we used the Moving average (Immediate) function in the Epoch average box.

At the baseline EEG registration of each second and subsequent session by alternating testing, we selected the frequency band and other corresponding components for the trained model that predicted the minimal deep hypnosis probability. This was a sign that the chosen components were at least properly predicting the wakefulness state. Further, we conducted a session with them to watch the Predictive curve.

**Figure S3.** Scenario-III in OpenVibe Designer. Each box contains its short functionality description on the left, except those already described in the previous scenarios


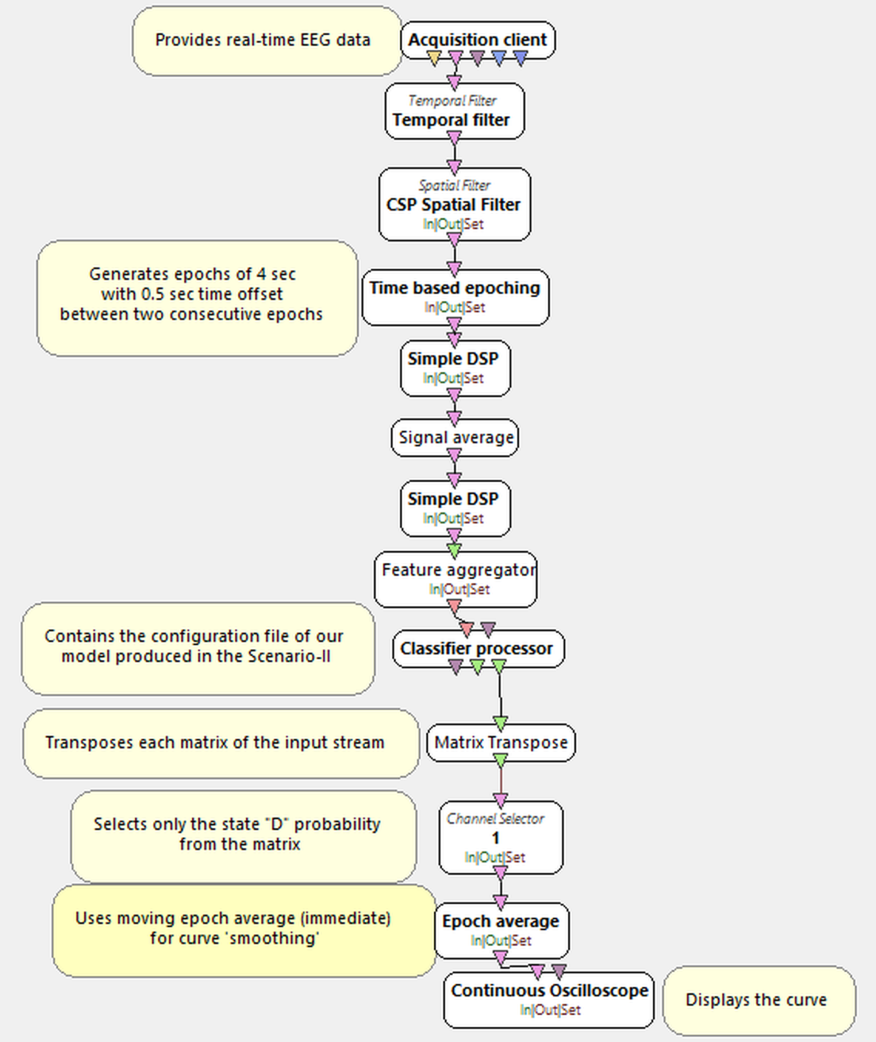


1. **Estimating the classification accuracy of the models based on new data (data from the second and subsequent sessions)**

The obtained EEG recordings of the second and subsequent sessions were labelled according to the same principle used for the first (calibration) sessions. Since the results of each second and subsequent session were already known to the hypnotherapist in the form of the Predictive curve, to eliminate bias in the file mark-up process, it was conducted by a specialist who did not attend those sessions and therefore had no prior knowledge of the predictive results obtained from them. Next, the marked file was passed through Scenario-IV (see Figure S4 below), which was almost completely the same as Scenario-III, but this labelled EEG was the data source in this case. Also, this scenario uses the Classifier Accuracy Measure box, which computes the accuracy of the model given the results from the classifier compared to the labels received. This is measured as the percentage of correctly classified epochs for the entire recording [18]. At this stage of our analysis, we tested the accuracy of four types of models for each of the second and subsequent sessions.

The training on marked-up data from each second and subsequent session resulted in obtaining their own (auxiliary) classification models. We did not use them further for real-time classification, but we put them in Scenario-IV and passed through that scenario the same data on which these models were trained. In this way, after each visit, we plotted a curve that reflected the dynamics of hypnotic depth in a given session as it actually occurred, i.e., as accurately as possible. We called it the Native curve. Then, the configurations of the Native and Predictive curves of the same sessions were visually compared to additionally assess how accurately the model trained on the first session data was able to reflect the actual picture of the subsequent ones.

**Figure S4.** Scenario-IV in OpenVibe Designer. Each box contains its short functionality description on the left, except those already described in the previous scenarios


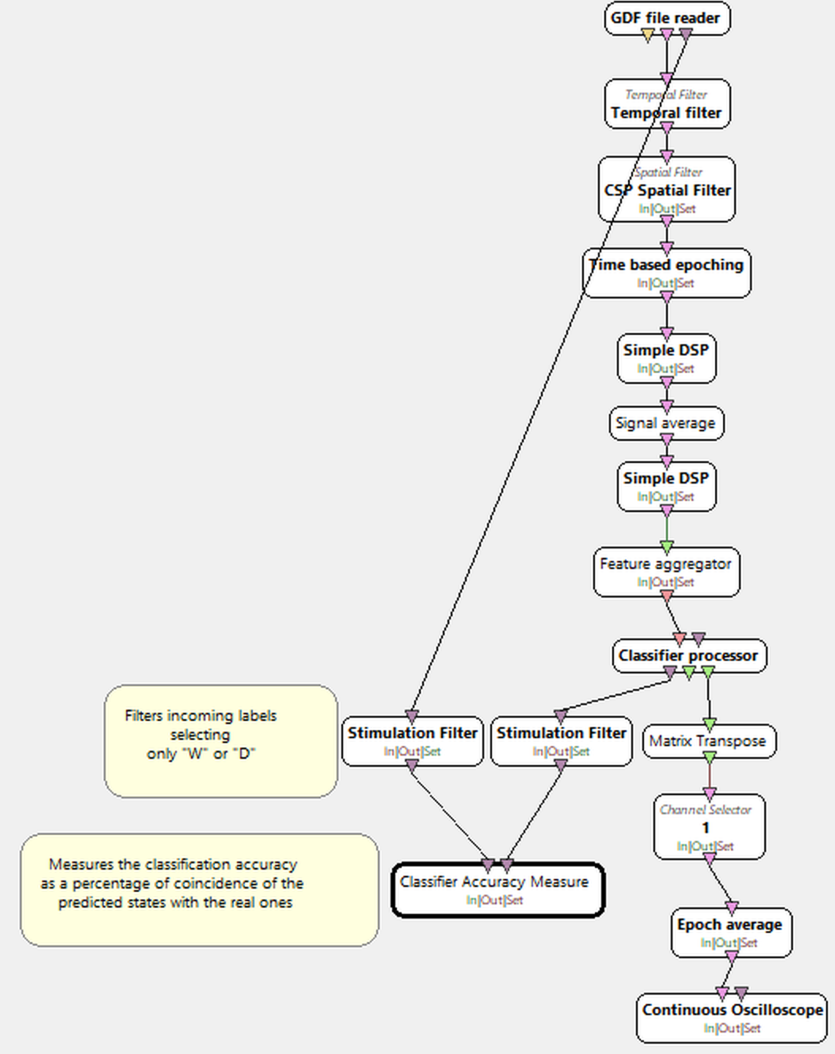


**References**

1. ​ Casiglia E, Finatti F, Tikhonoff V, Stabile MR, Mitolo M, Gasparotti F, et al. Granone's plastic monoideism demonstrated by functional magnetic resonance imaging (fMRI). Psychology. 2019;10(04):434–48.
2. ​ Casiglia E, Finatti F, Tikhonoff V, Stabile MR, Mitolo M, Albertini F, et al. Mechanisms of hypnotic analgesia explaned by functional magnetic resonance (fMRI). Int J Clin Exp Hypn. 2020;68(1):1–15.
3. Casiglia E, Tikhonoff V, Albertini F, Lapenta AM, Gasparotti F, Finatti F, et al. The mysterious hypnotic analgesia: experimental evidences. Psychology. 2018;09(08):1935–56.
4. ​Almeida-Marques FX, Sánchez-Blanco J, Cano-García FJ. Hypnosis is more effective than clinical interviews. Int J Clin Exp Hypn. 2018;66(1):3–18.
5. Diamond SG, Davis OC, Howe RD. Heart-rate variability as a quantitative measure of hypnotic depth. Int J of Clin and Exp Hypn. 2007;56(1):1–18.
6. Dunham CM, Burger AJ, Hileman BM, Chance EA, Hutchinson AE. Bispectral index alterations and associations with autonomic changes during hypnosis in trauma center researchers: formative evaluation study. JMIR Form Res. 2021;5(5):e24044.
7. Excoffier J, Pichot V, Cantais A, Mory O, Roche F, Patural H, Chouchou F. Autonomic cardiac reactivity to painful procedures under hypnosis in pediatric emergencies: a feasibility study. Am J Clin Hypn. 2020;62(3):267–281.
8. Kasos K, Csirmaz L, Vikor F, Zimonyi S, Varga K, Szekely A. Electrodermal correlates of hypnosis: current developments. OBM Integr Complem Med. 2020;5(2):1–20.
9. Kekecs Z, Szekely A, Varga K. Alterations in electrodermal activity and cardiac parasympathetic tone during hypnosis. Psychophysiology. 2016;53(2):268–77.
10. Casiglia E, Finatti F, Gasparotti F, Stabile MR, Mitolo M, Albertini F, et al. Functional magnetic resonance imaging demonstrates that hypnosis is conscious and voluntary. Psychology. 2018;09(07):1571–81.
11. Barrett D. Hypnosis and empathy: a complex relationship. Am J Clin Hypn. 2016;58(3):238–50.
12. ​Barrett D. Fantasizers and dissociaters: two types of high hypnotizables, two different imagery styles. In: Kunzendorf RG, Spanos NP, Wallace B, editors. Hypnosis and imagination. 1st ed. Routledge; 1996. p. 123–35.
13. Barrett D. Fantasizers and dissociaters: data on two distinct subgroups of deep trance subjects. Psychol Rep. 1992;71(3):1011–4
14. Aydemir Ö. Common spatial pattern-based feature extraction from the best time segment of BCI data. Turk J Elec Eng Comp Sci. 2016;24(5):3976–86.
15. Blankertz B, Tomioka R, Lemm S, Kawanabe M, Muller K. Optimizing spatial filters for robust EEG single-trial analysis. IEEE Signal Process Mag. 2008;25(1):41–56.
16. Bird JJ, Buckingham CD, Ekárt A, Faria DR. Mental emotional sentiment classification with an EEG-based brain-machine interface. 2019. http://jordanjamesbird.com/publications/Mental-Emotional-Sentiment-Classification-with-an-EEG-based-Brain-machine-Interface.pdf. Accessed 5 May 2023.
17. ​​Ramoser H, Muller-Gerking J, Pfurtscheller G. Optimal spatial filtering of single trial EEG during imagined hand movement. IEEE Trans Rehabil Eng. 2000;8(4):441–6.
18. Kohavi R, Provost F. Glossary of terms. Special issue of applications of machine learning and the knowledge discovery process. Mach Learn. 1998;30(2/3):271–4.
