# Supplementary material for: Real-time assessment of hypnotic depth, using an EEG-based brain-computer interface: a preliminary study
Source: BMC Res Notes. 2023 Oct 24;16:288. doi: 10.1186/s13104-023-06553-2 (PMC10599062; doi:10.1186/s13104-023-06553-2)
Supplement: Supplementary file 2 — Supplementary Material 2 (“Supplements B”) [file 13104_2023_6553_MOESM2_ESM.docx]

**Supplements B**

Topographic maps, displaying the changes in the power of different rhythms for different localizations while achieving deep hypnosis. Changes are displayed in colour according to the graduation of a nearby colour scale. The colour scale is in uV². The standard bands are Delta (1.5-4 Hz), Theta (4-8 Hz), Alpha (8-12 Hz), Sensory-motor or Low beta (12-15 Hz), Beta1 (15-18 Hz), Beta2 (18-25 Hz) and Gamma (25-45 Hz). The data for all included patients and sessions is presented. The detailed analysis is presented below the maps

**Patient A, session 1**


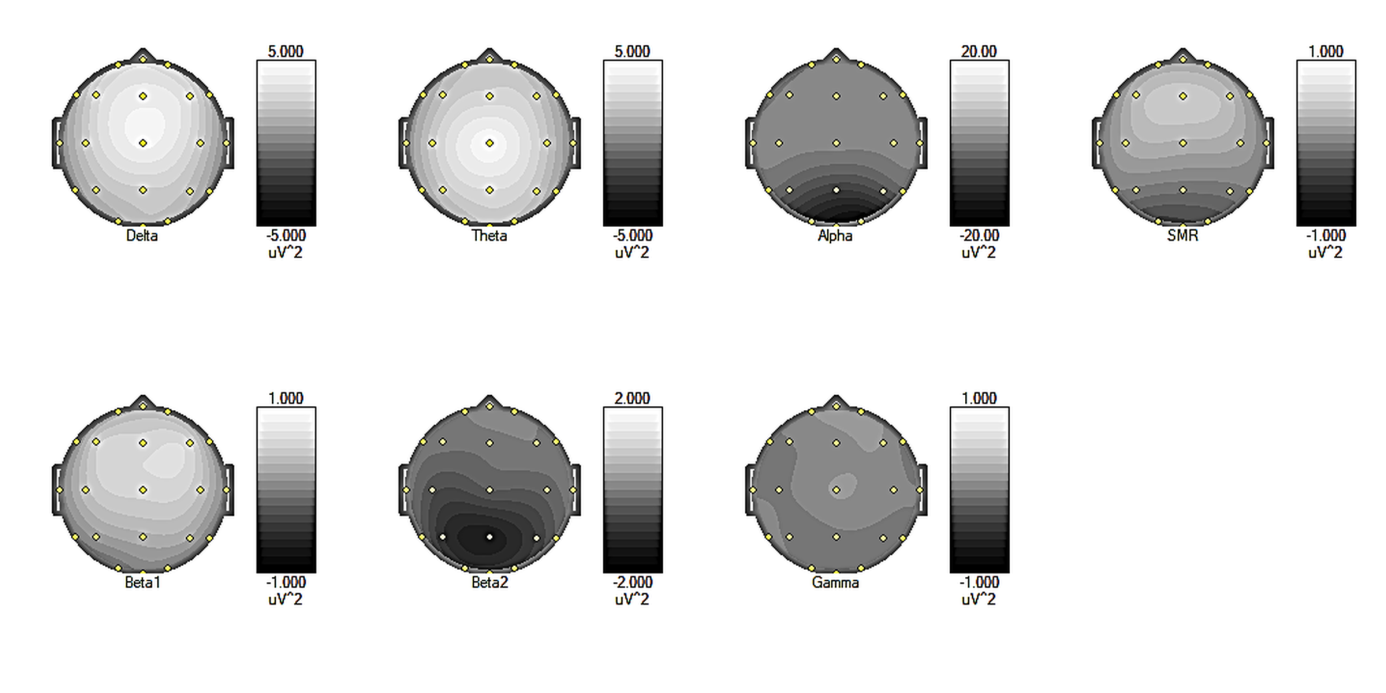


**Patient A, session 2**


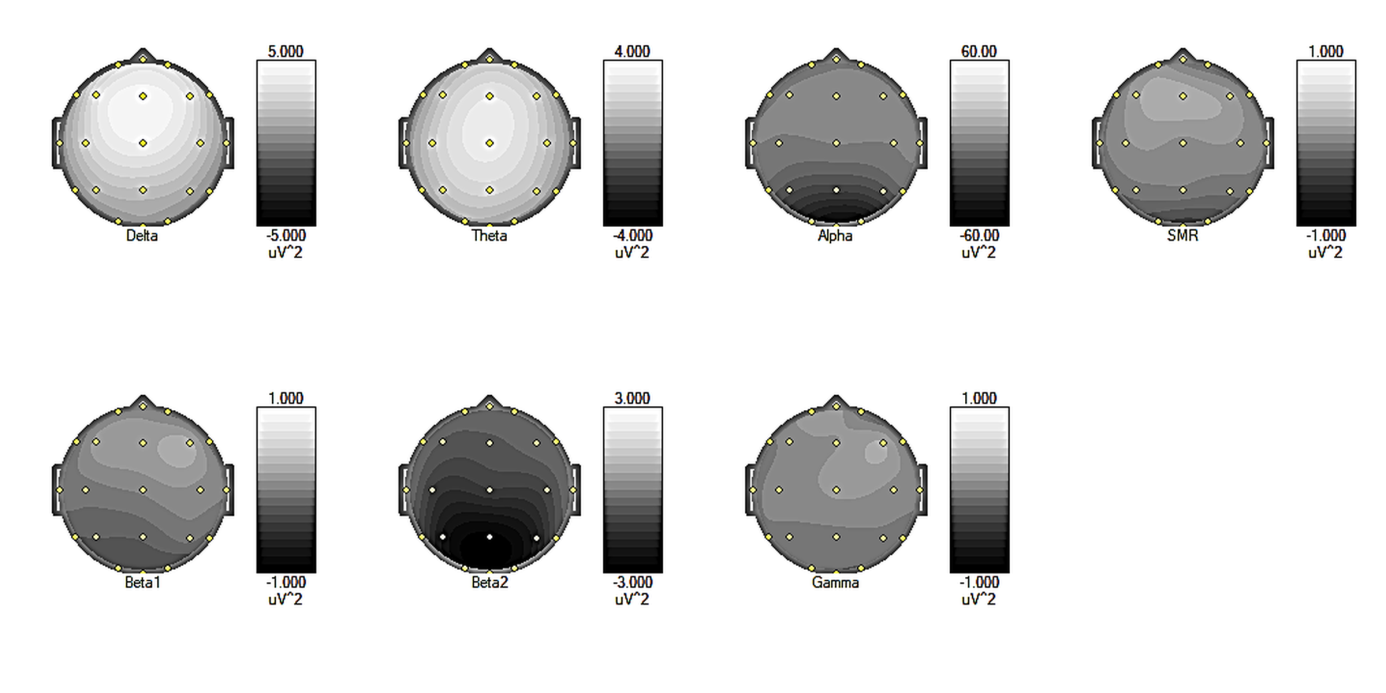


**Patient A, session 3**


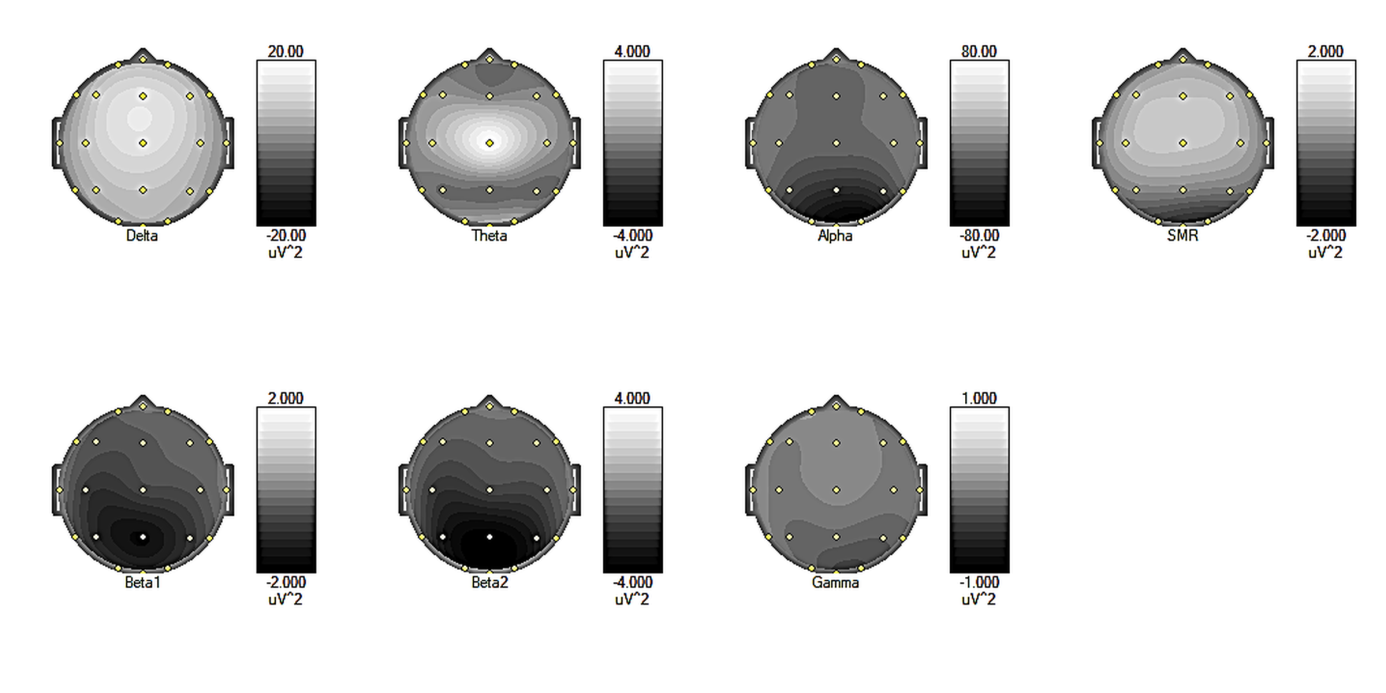


**Patient A, session 4**


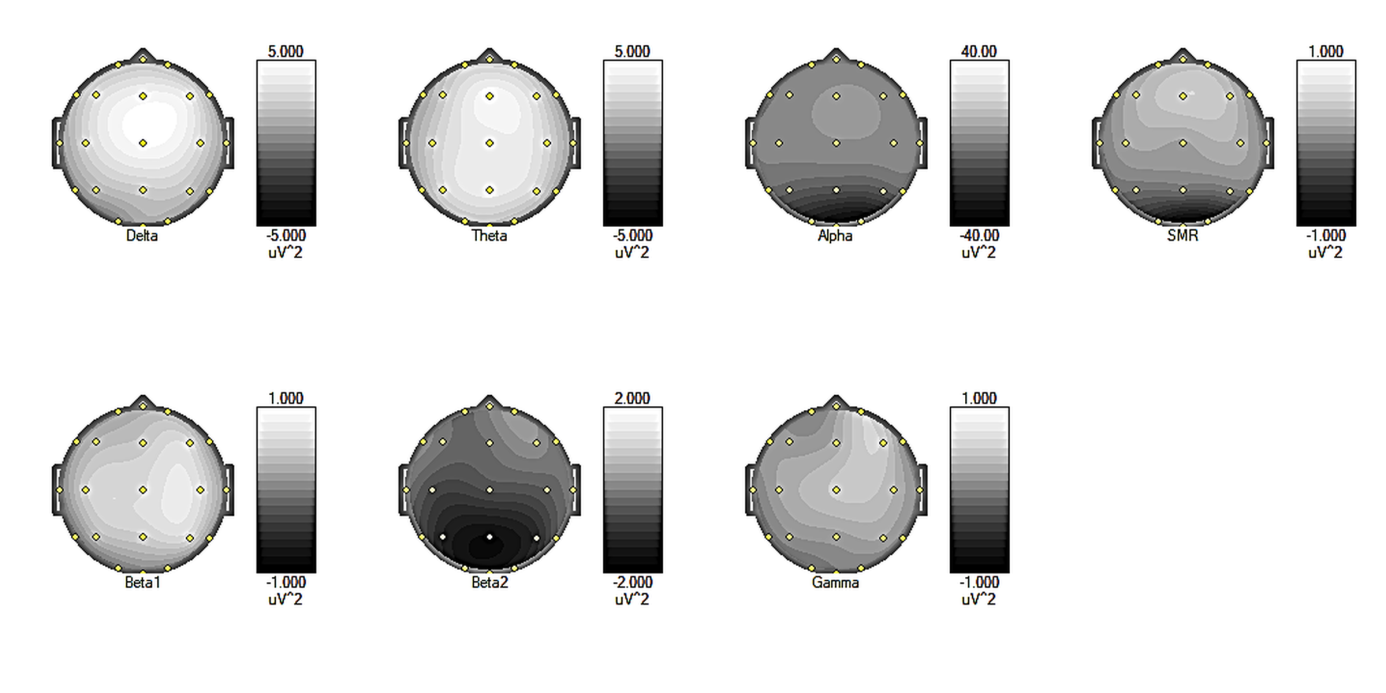


**Patient A, session 5**


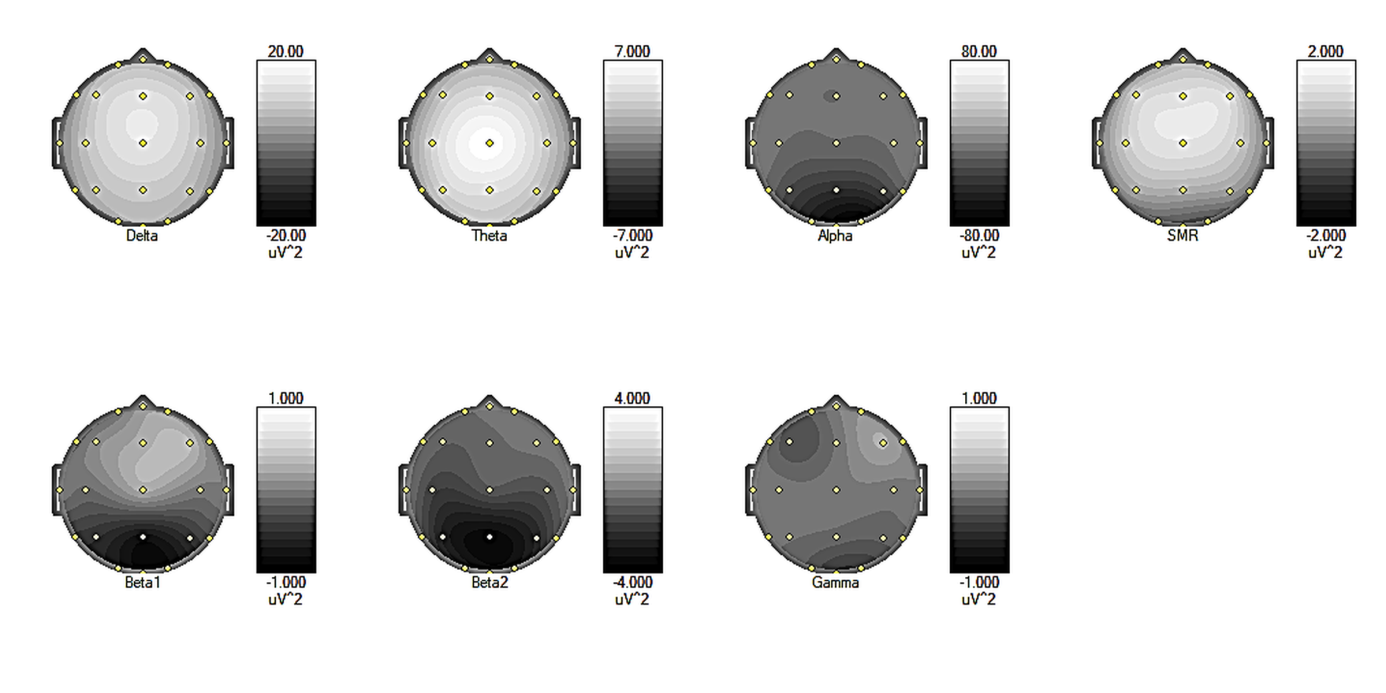


**Patient A, session 6**


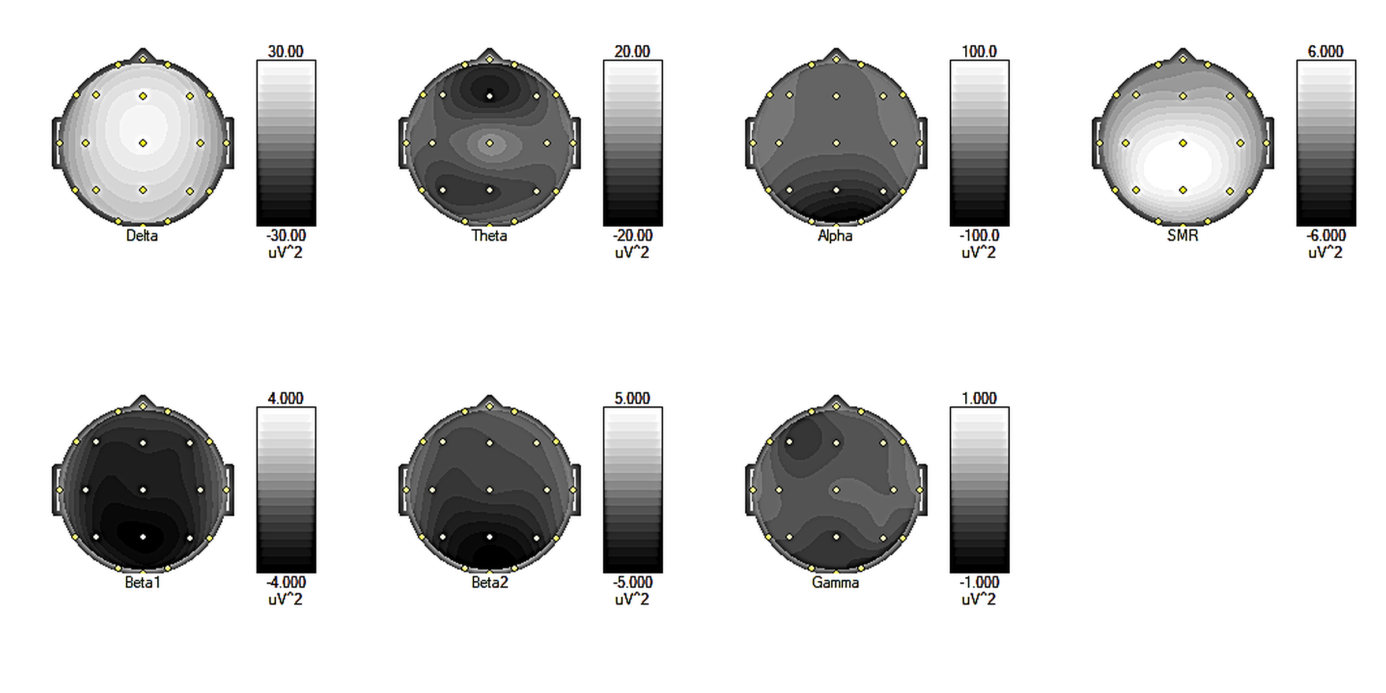


**Patient A, session 7**


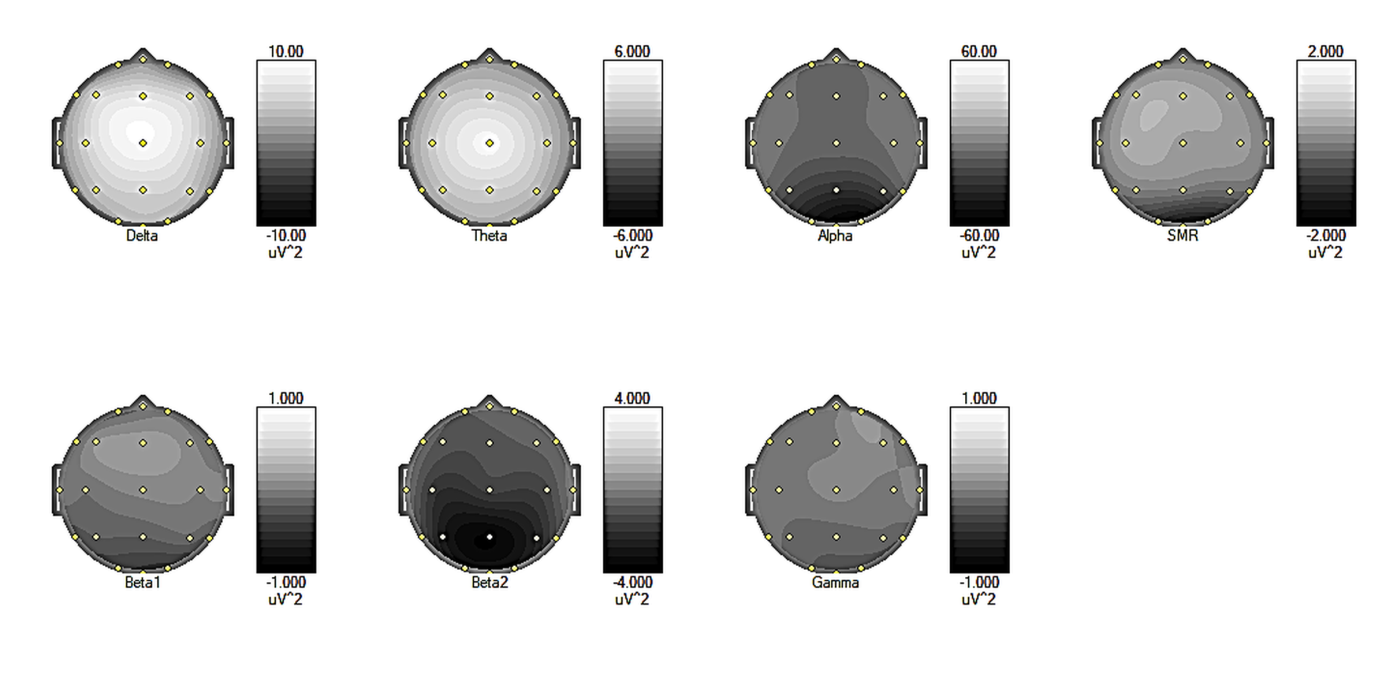


**Patient E, session 1**


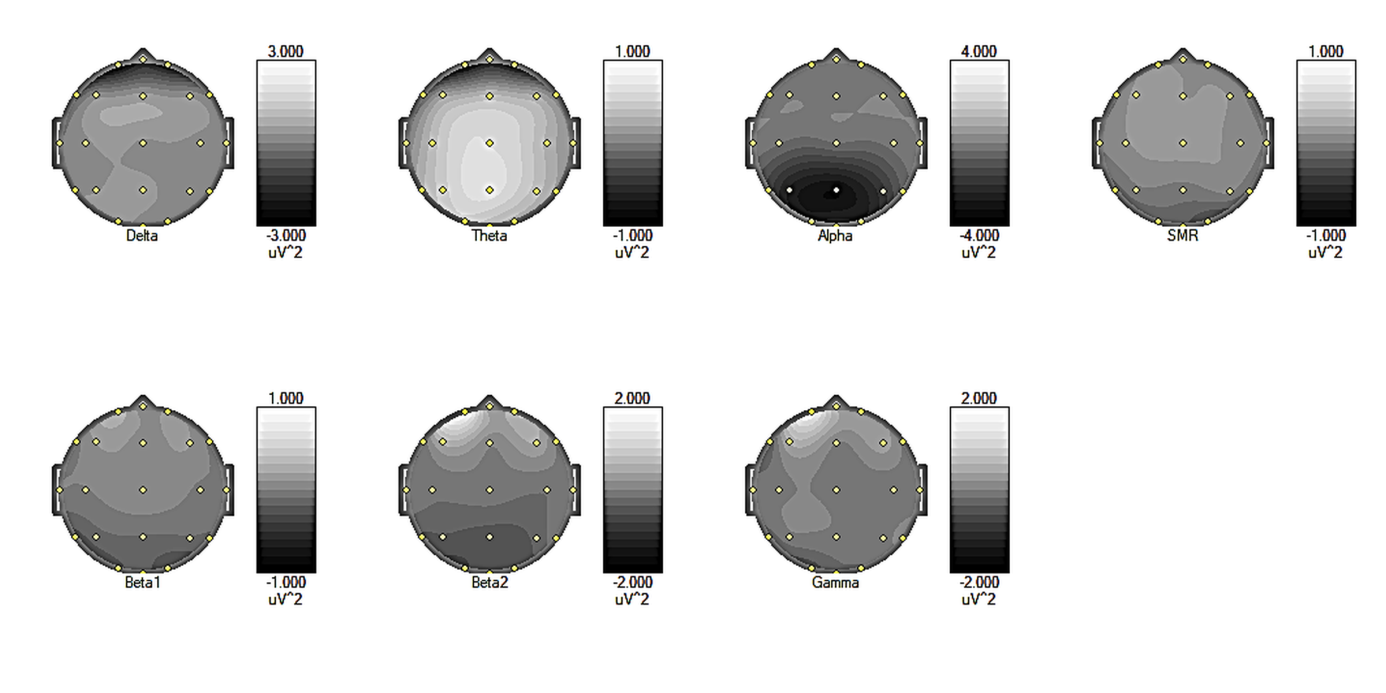


**Patient E, session 2**


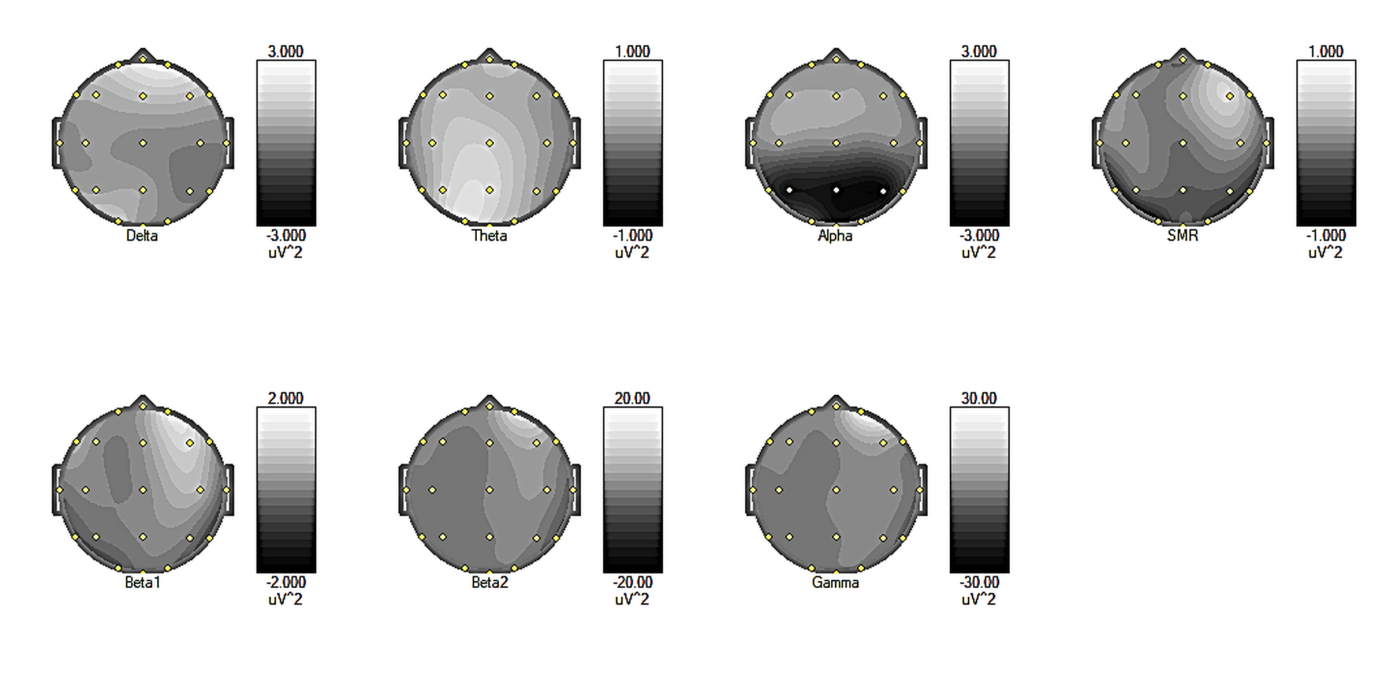


**Patient E, session 3**


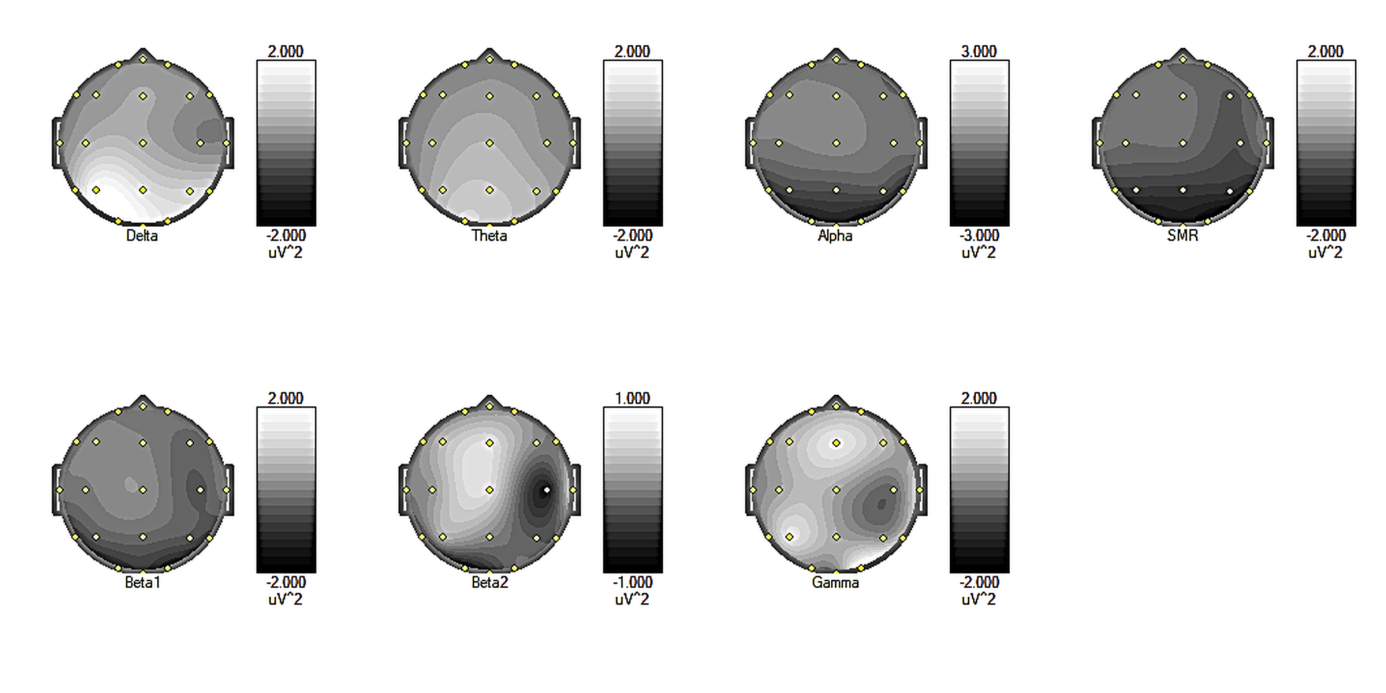


**Patient E, session 4**


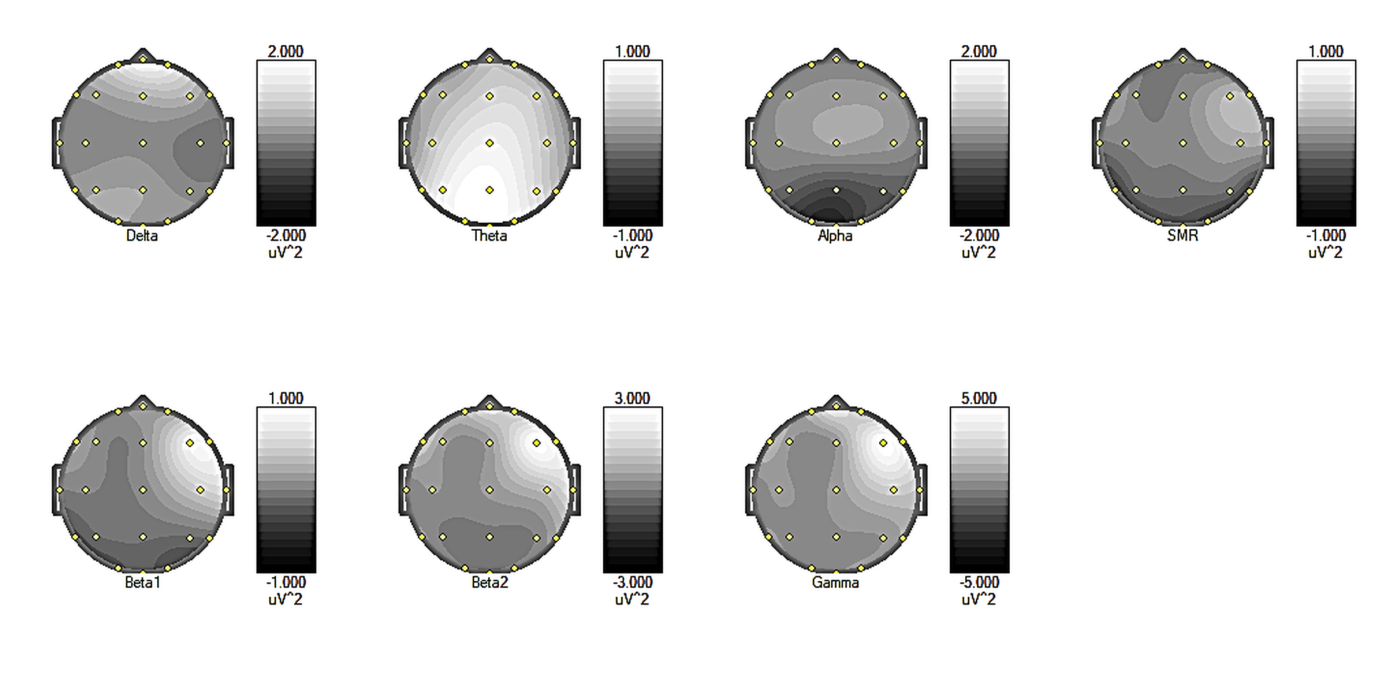


**Patient E, session 6 (the 5^th^ was excluded)**


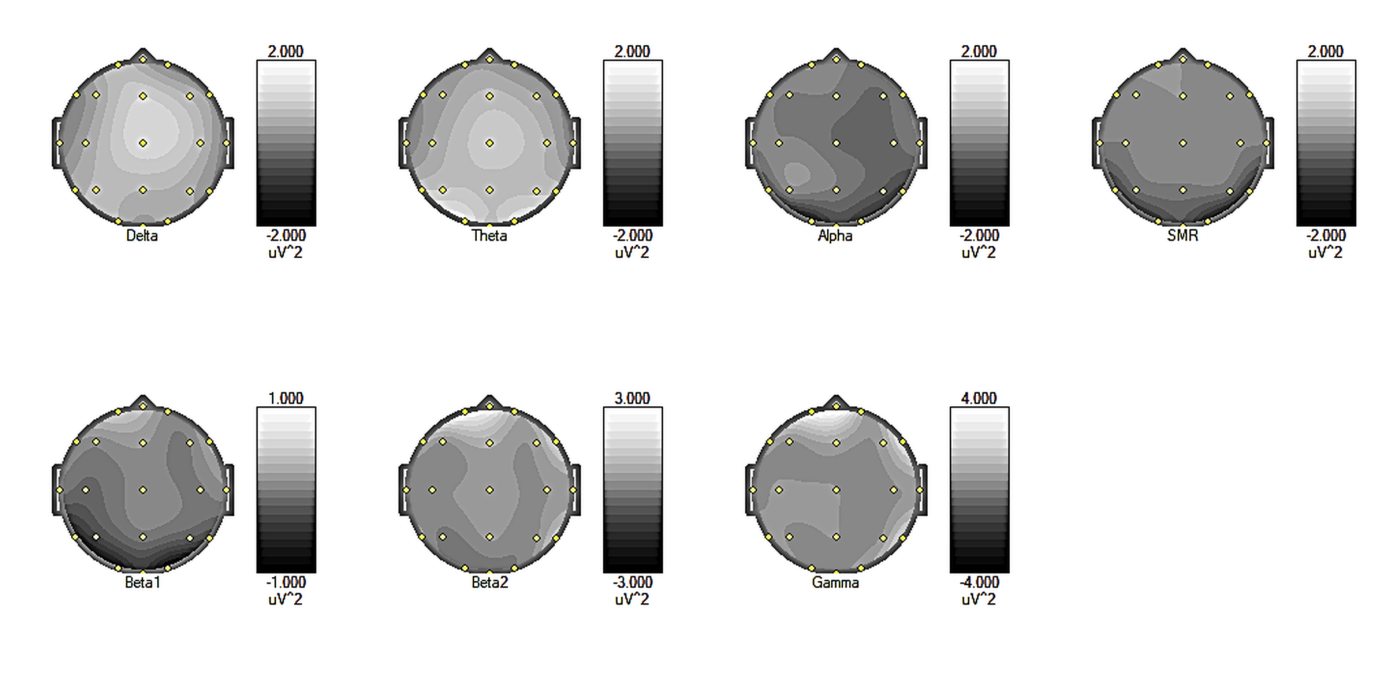


**Patient G, session 1**


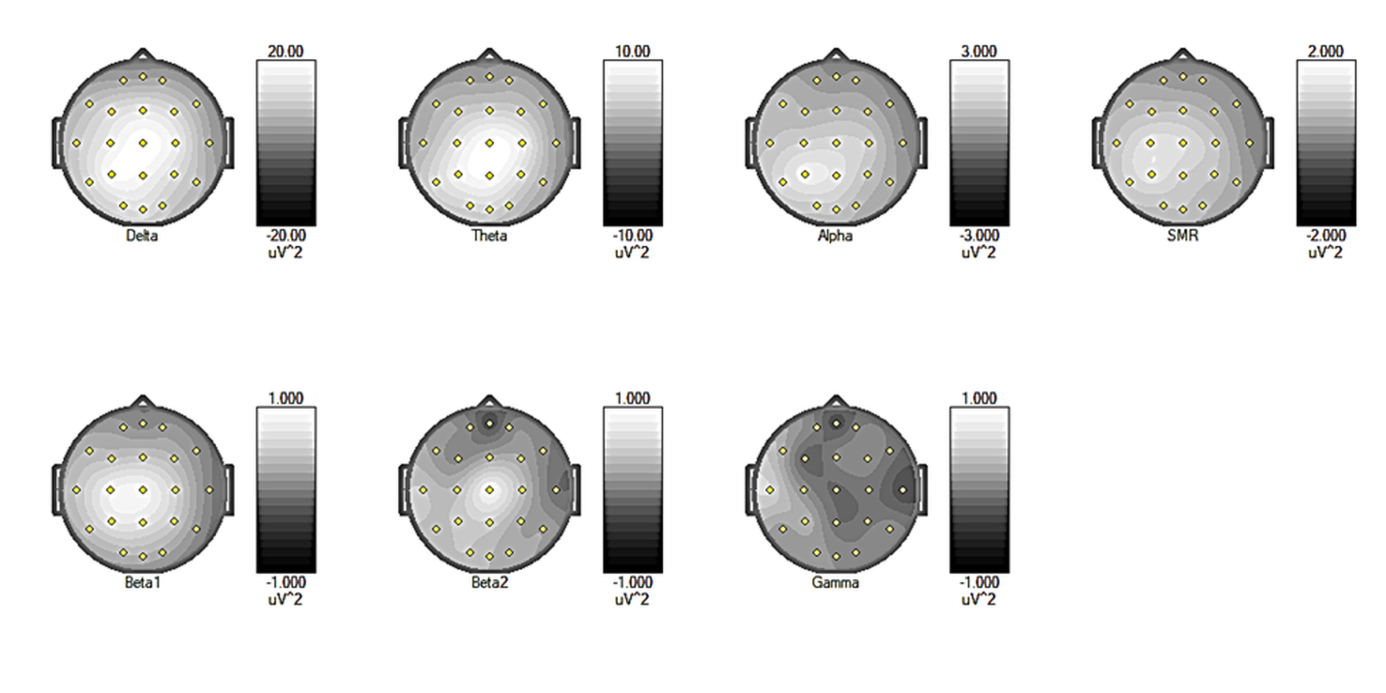


**Patient G, session 2**


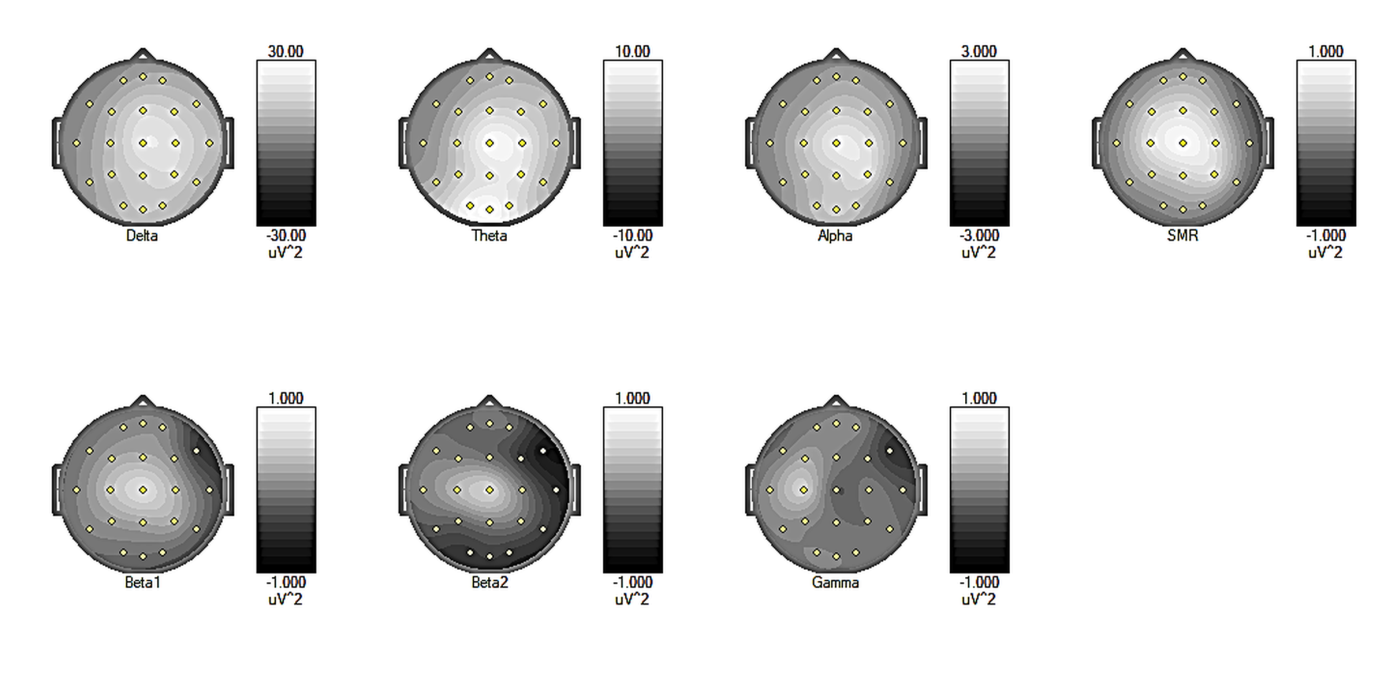


**Patient G, session 3**


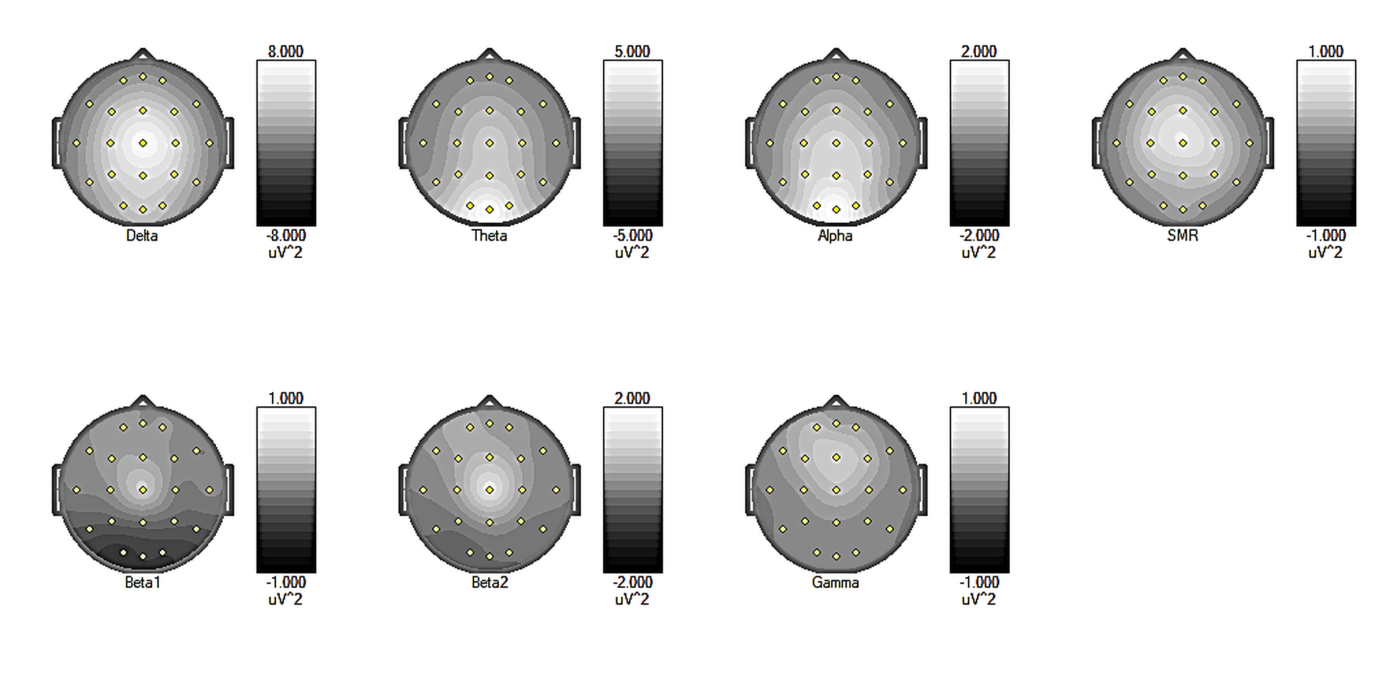


**Patient G, session 4**


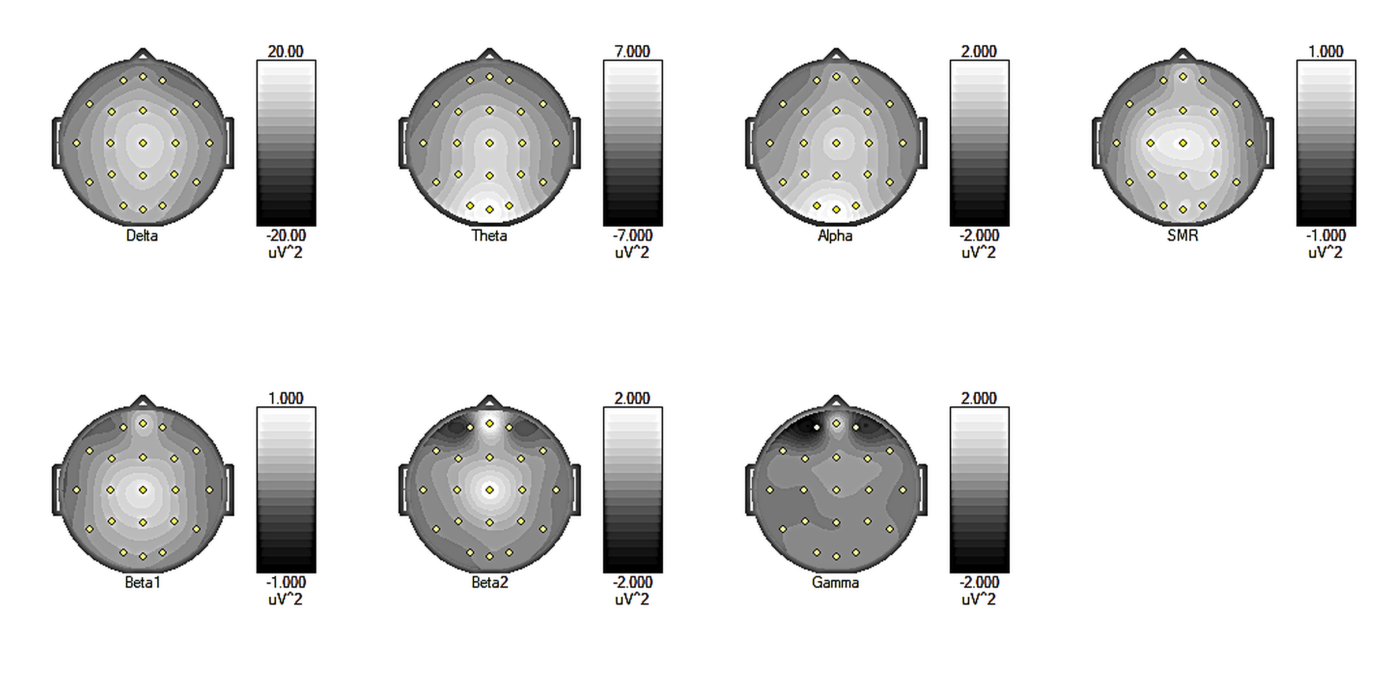


**Patient S, session 1**


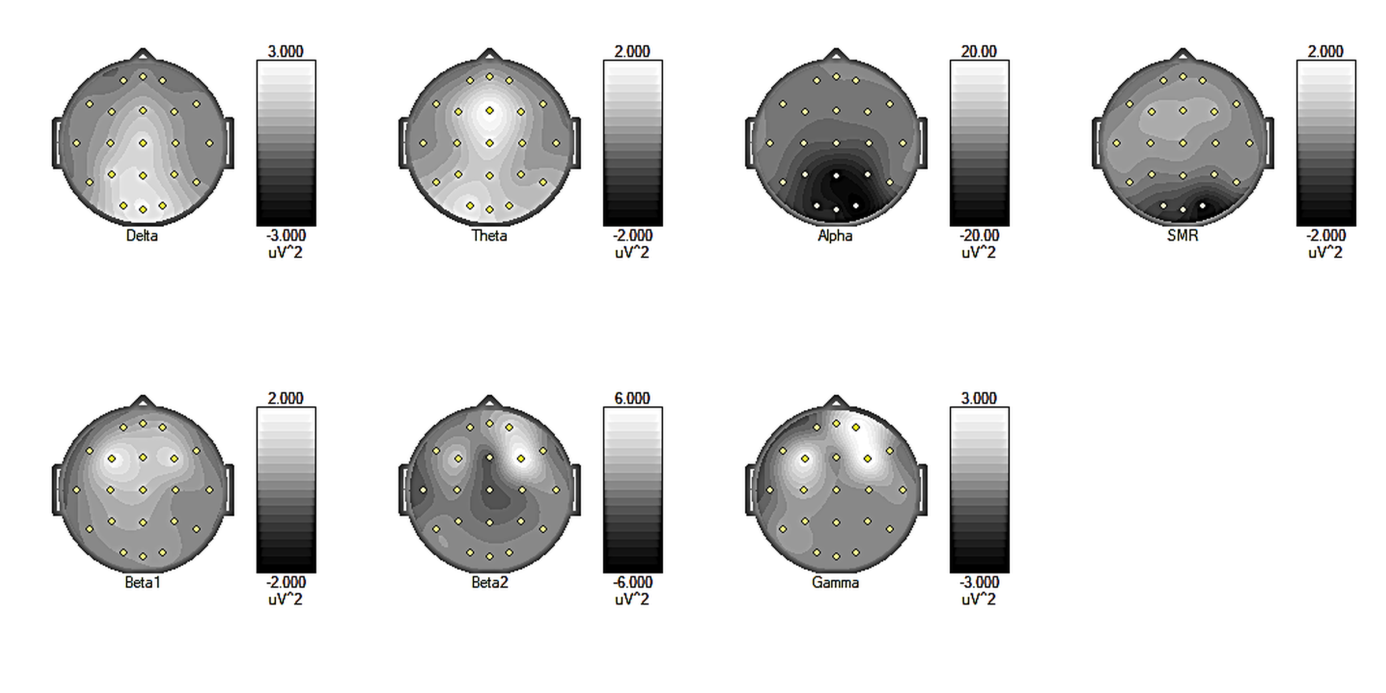


**Patient S, session 2**


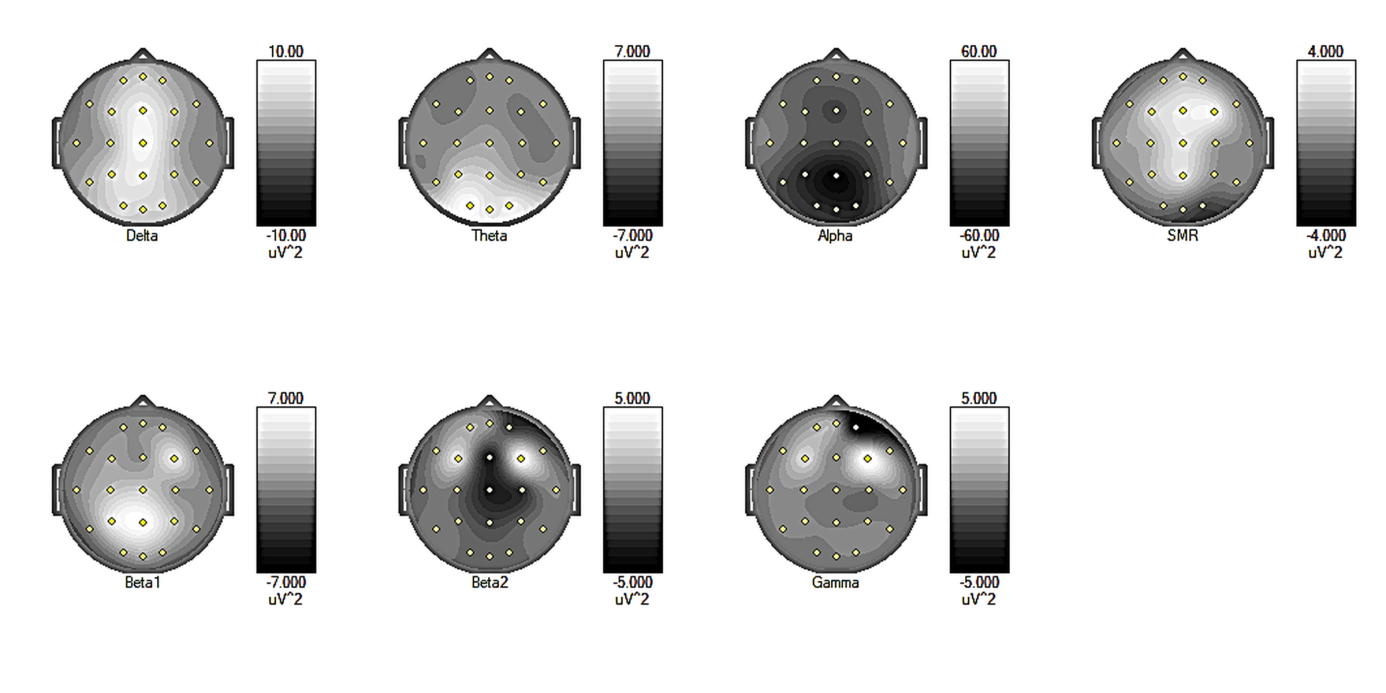


**Patient S, session 3**


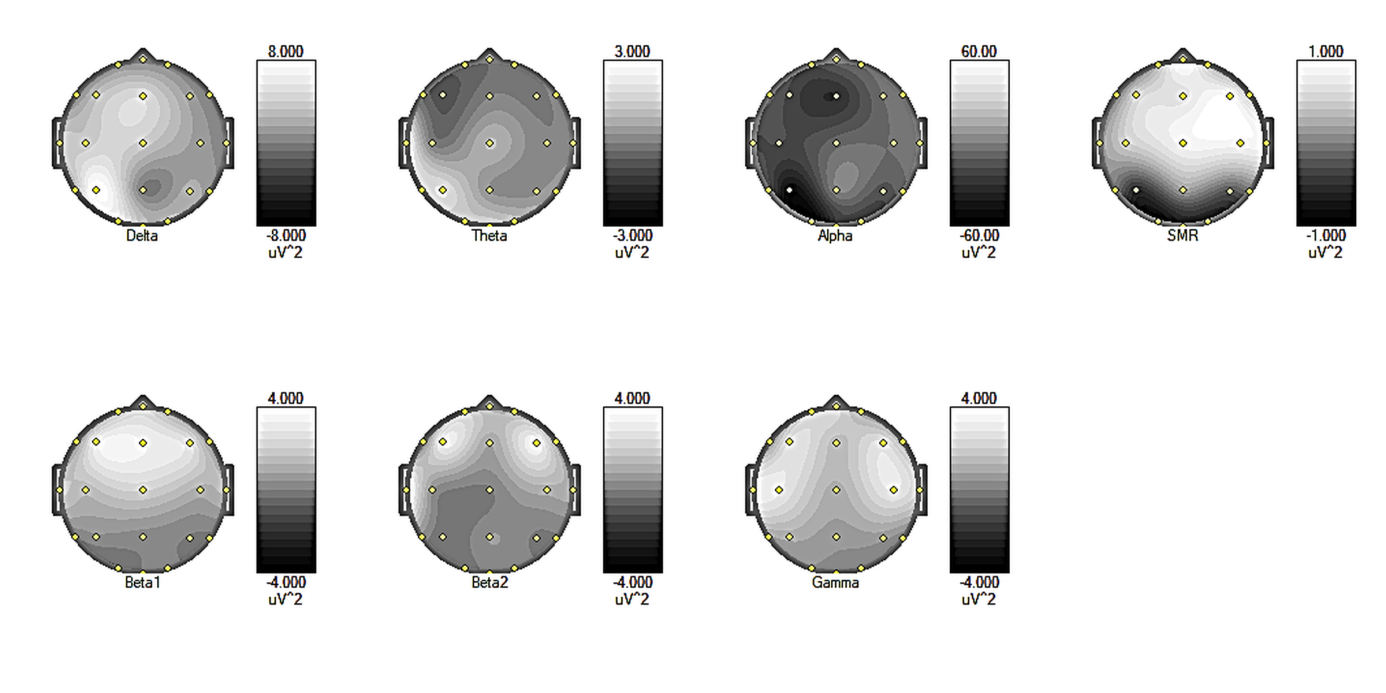


**Patient O, session 1**


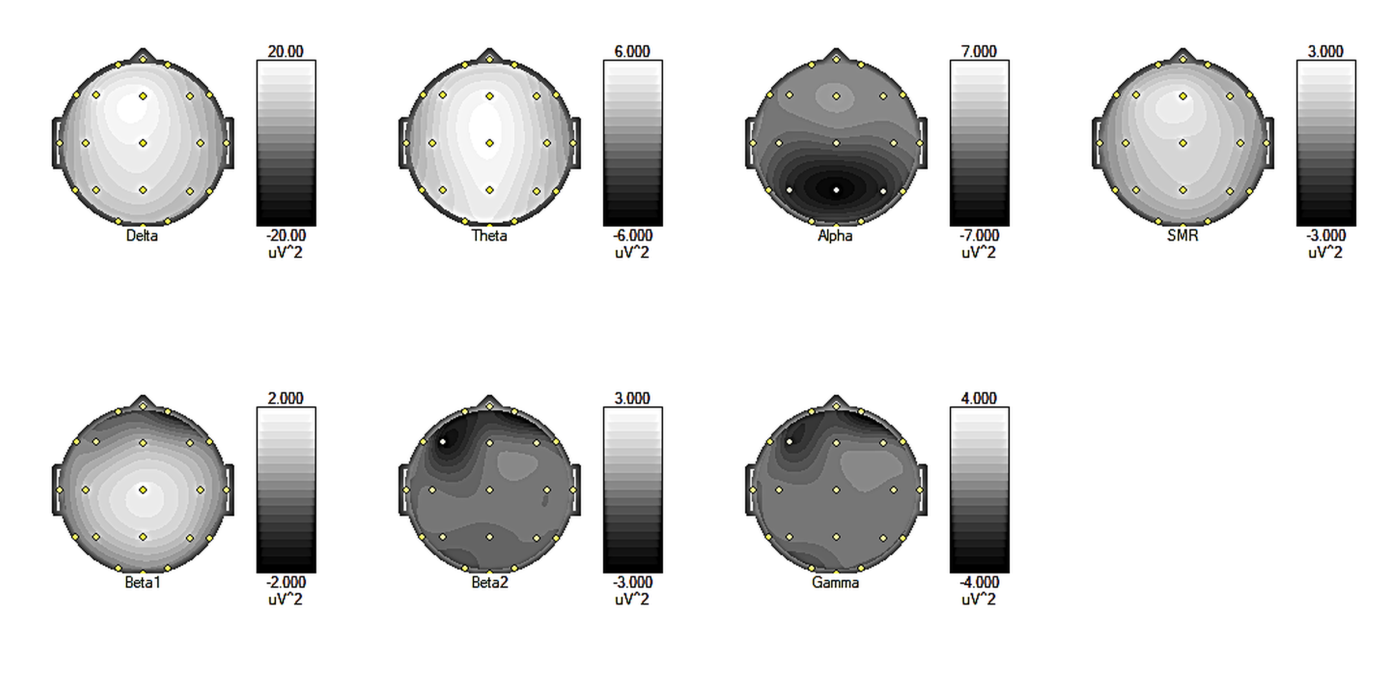


**Patient O, session 2**


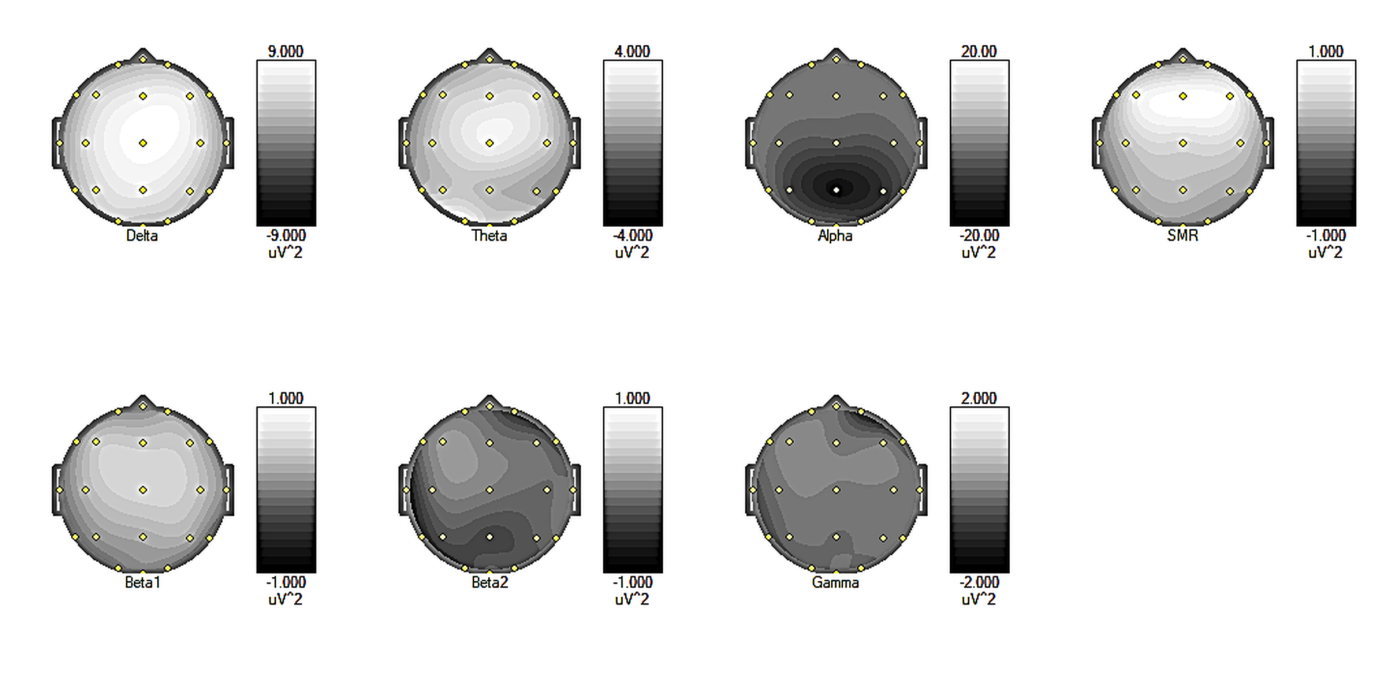


**Patient N, session 1**


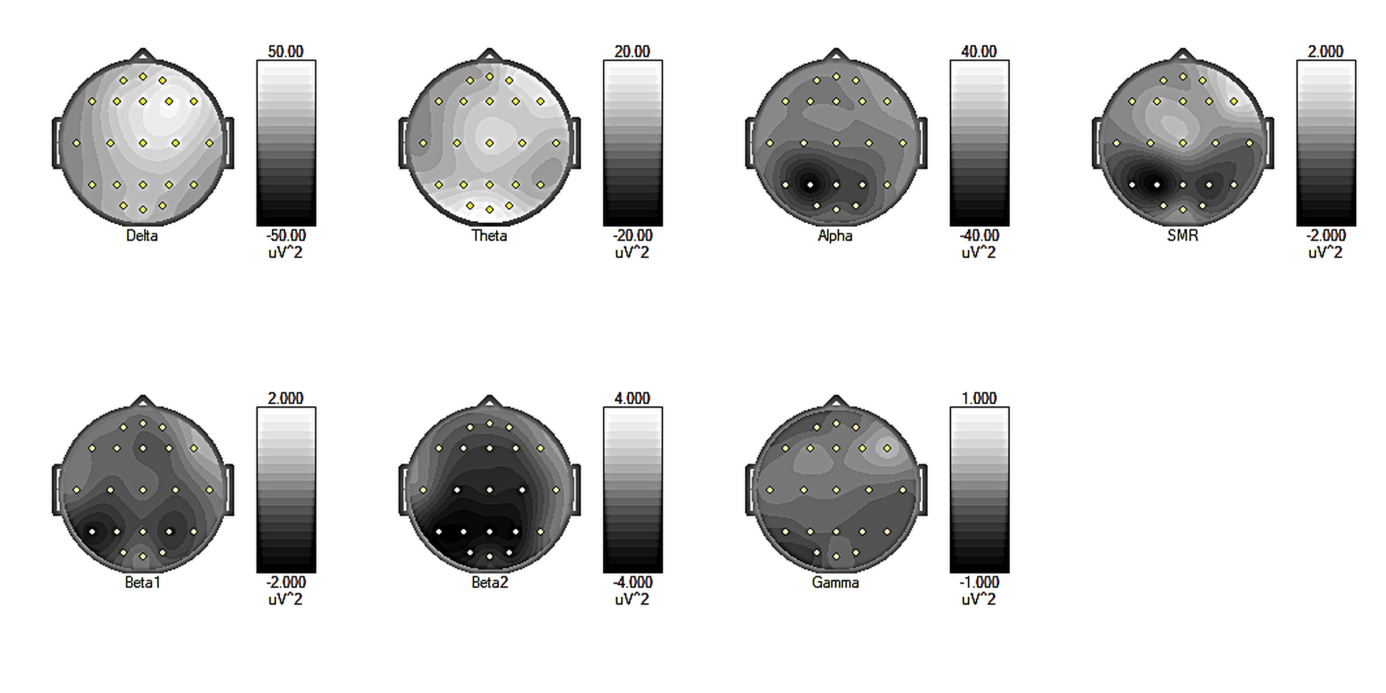


**Patient N, session 2**


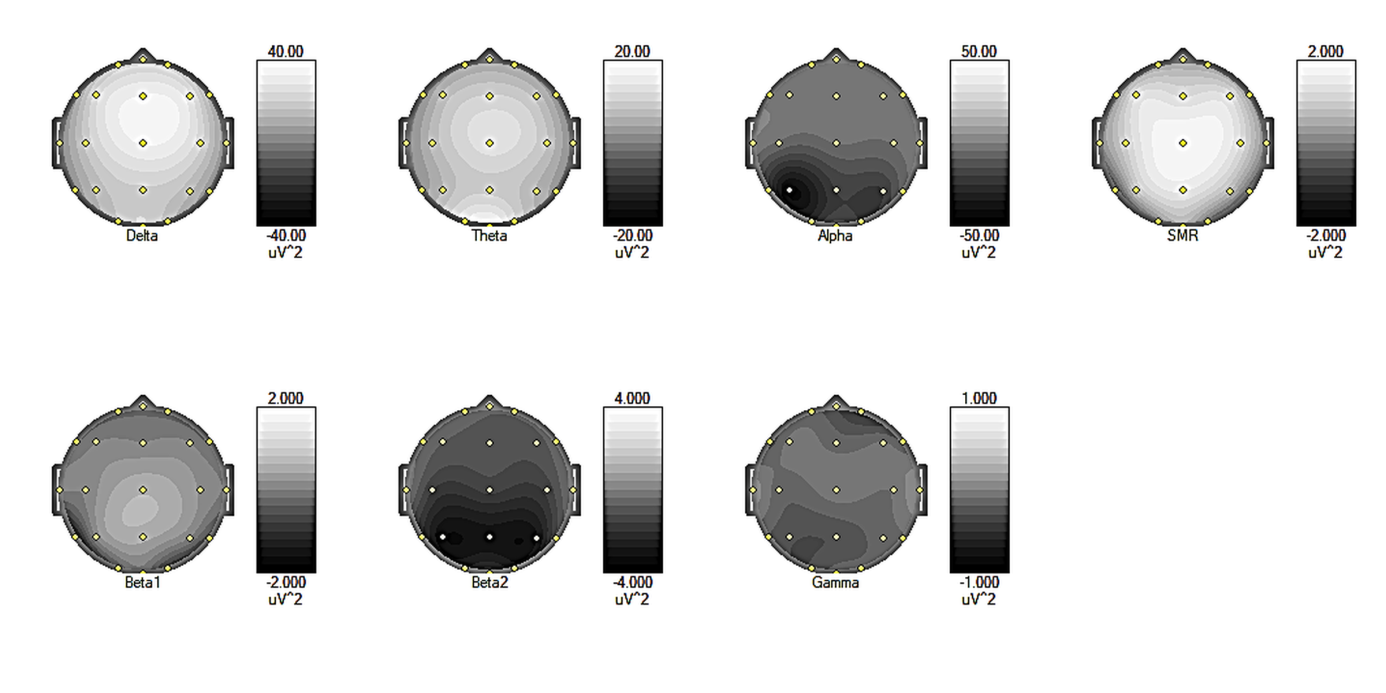


**Patient V, session 1**


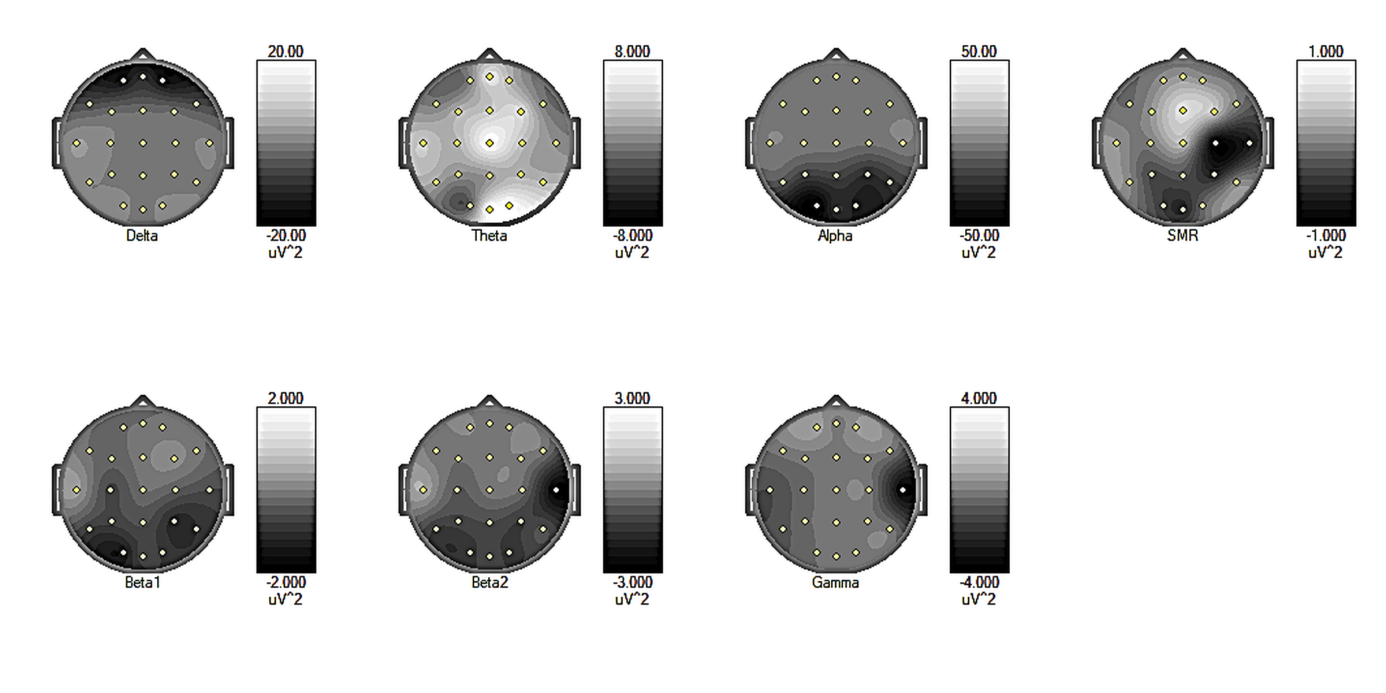


**Patient V, session 2**


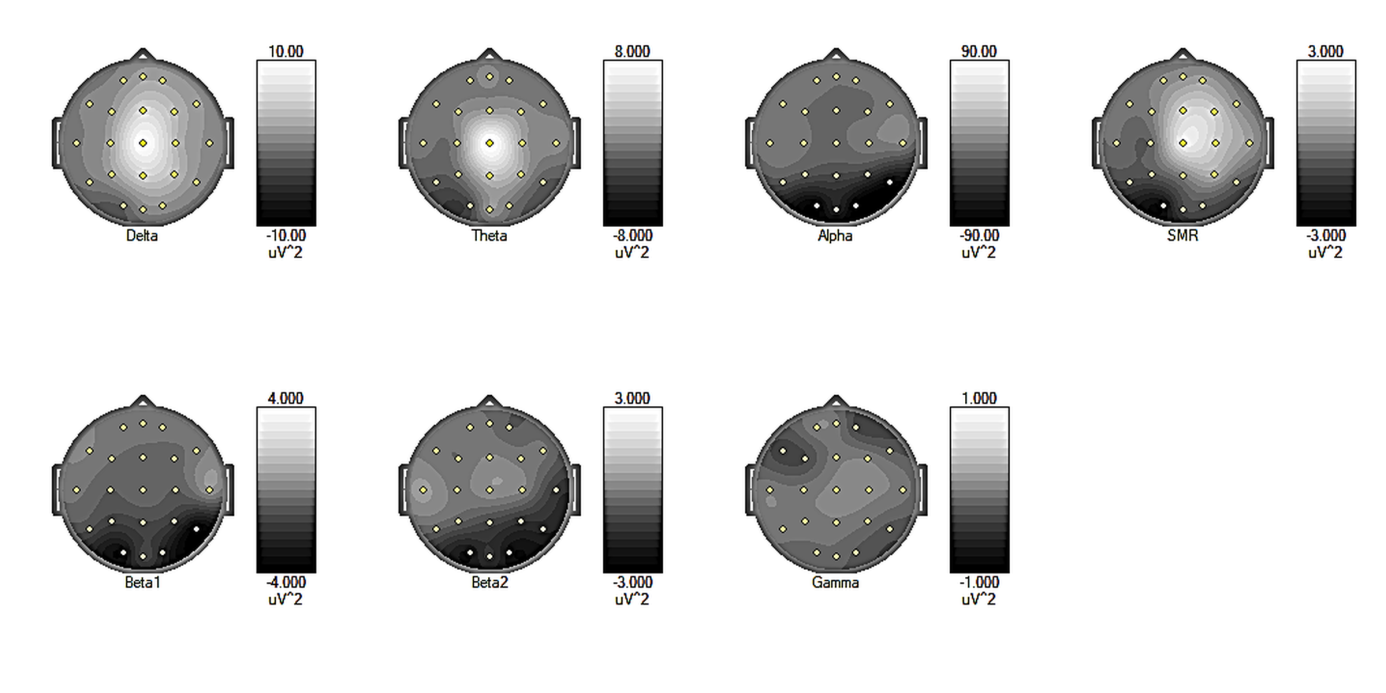


**Patient C, session 1**


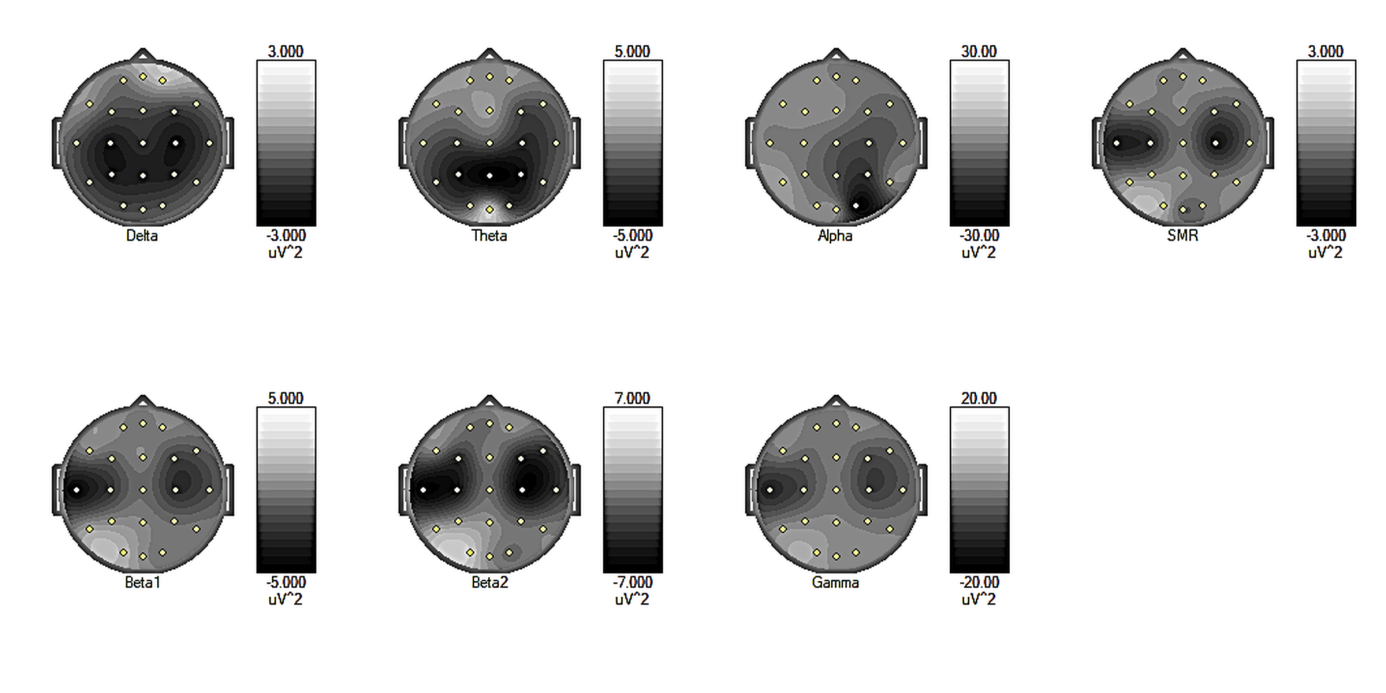


**Patient C, session 2**


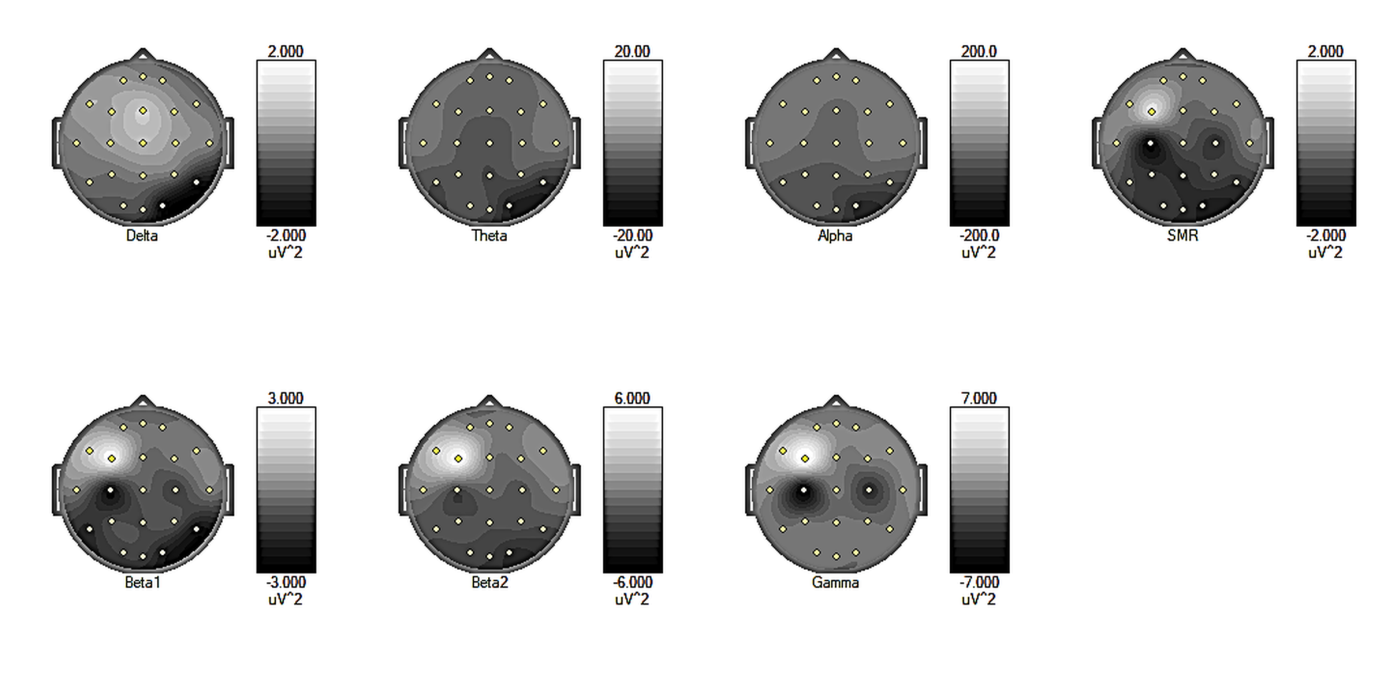


The results of this analysis provide tentative (and qualitative) information on what rhythms in which brain areas were changing after achieving deep hypnosis in a particular session. For example, as we can see from the topographic maps of Patient A, his transition to a deep hypnotic state is associated with a very large decrease in the alpha range activity, some decrease in the beta-2 rhythm in the occipital regions, a noticeable increase in theta, and even slower activity in the middle and mid-frontal regions, as well as some increase in the sensorimotor and beta-1 rhythms in the frontal region. This combination (i.e., estimated individual hypnotic patterns) tends to be observed in each of his sessions. Other patients' maps demonstrate some similar changes: slow-wave activity increases in different areas of their brain, which is consistent with the literature on the electrophysiological correlates of hypnosis. Nevertheless, they also show quite clear differences between patients. For example, unlike all the other participants in the study, Patient G did not demonstrate a decrease in the alpha rhythm in the occipital region; on the contrary, she showed an increase along with an increase in the power of slower rhythms. In addition, we can see from the maps that the estimated patterns of deep hypnosis in a given patient show a degree of stability and tend to reproduce from session to session. The example of Patient E demonstrates that this reproducibility could be observed even if more than a year passed between the measurements.

Thus, based on the analysis of the topographic maps of all 27 hypnosis sessions, we could tentatively infer that: a) the estimated EEG patterns of deep hypnosis tend to be generally stable from session to session in a given patient, which suggests that b) the model trained could predict the desired states correctly in each subsequent session. This analysis showed as well that c) although the deep state patterns might have similarities among patients, there are also differences between individuals. Therefore, a uniform approach for all people to "measure" hypnotic depth may probably yield more rough results than an individualised approach. These points suggest that machine learning could potentially apply to hypnosis.
