# Supplementary material for: Real-time assessment of hypnotic depth, using an EEG-based brain-computer interface: a preliminary study
Source: BMC Res Notes. 2023 Oct 24;16:288. doi: 10.1186/s13104-023-06553-2 (PMC10599062; doi:10.1186/s13104-023-06553-2)
Supplement: Supplementary file 3 — Supplementary Material 3 (“Supplements C”) [file 13104_2023_6553_MOESM3_ESM.docx]

**Supplements C**

**The pairs of the Predictive (upper) and Native (lower) curves for each patient for the second and subsequent sessions**

To be able to demonstrate the Predictive curves, we played them back with Scenario-IV (as with the Native curves) using recordings of the corresponding sessions. The screenshots of the Continuous Oscilloscope were taken at the end of the playbacks.

The frequency band used to plot the Predictive and Native curves is indicated here for each patient. The band that provided a high average classification accuracy for each patient was selected. For the convenience of displaying data over a large time interval, the curves are smoothed using the Moving Epoch Average (Immediate) function in the Epoch Average box. The number of 4-s epochs with an overlap of 0.5 s used for averaging is indicated for each pair. A detailed analytical description using an individual case of how the Predictive curve was generally able to reflect the phenomenology of a session is presented below the figures.

**Patient A**, the band used is 1.5-14 Hz

The 2^nd^ session: the number of epochs for averaging is 100


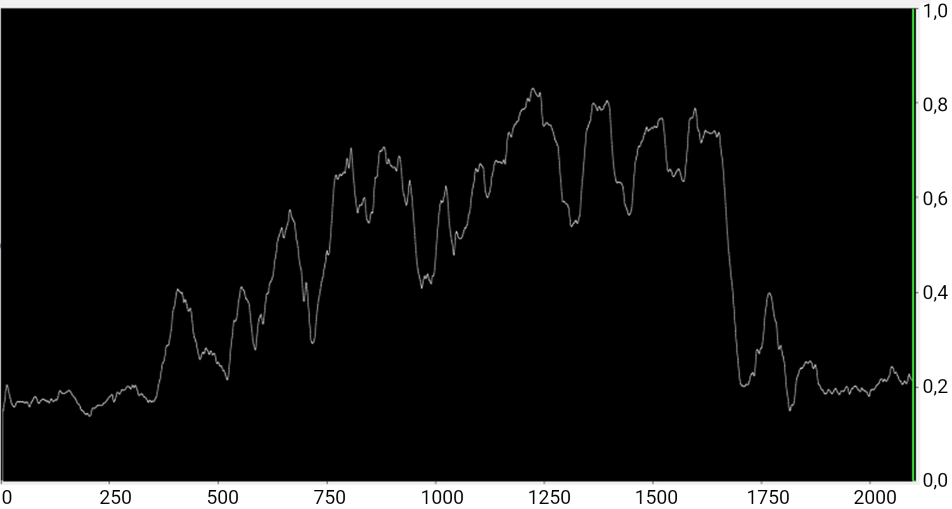


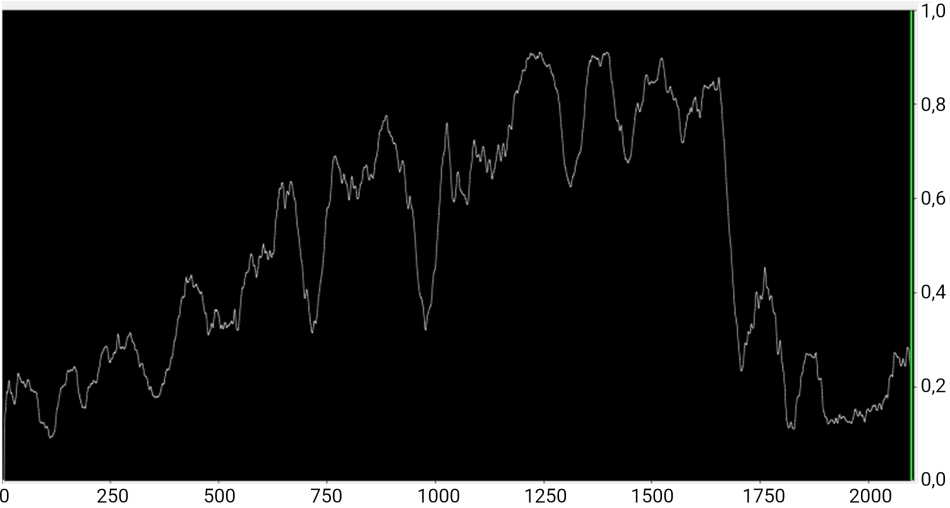


The 3^rd^ session: the number of epochs for averaging is 50


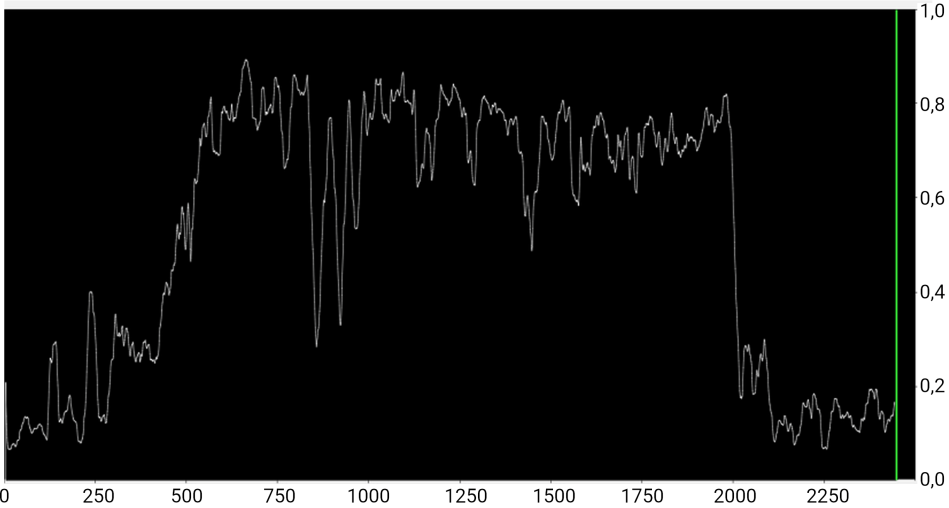


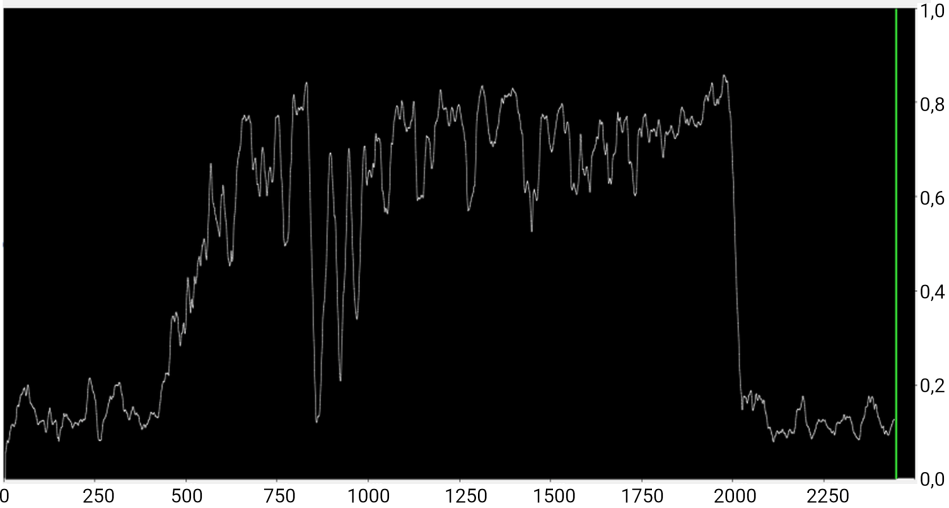


The 4^th^ session: the number of epochs for averaging is 100


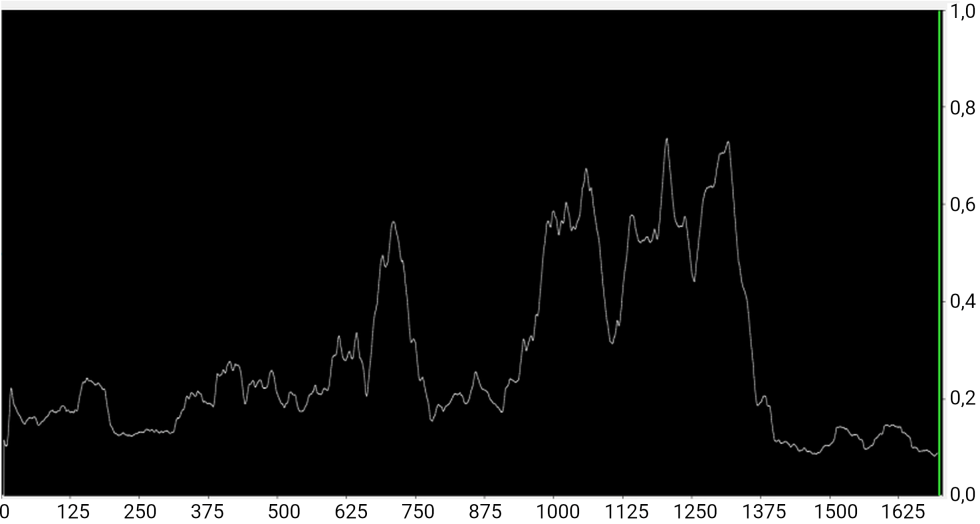


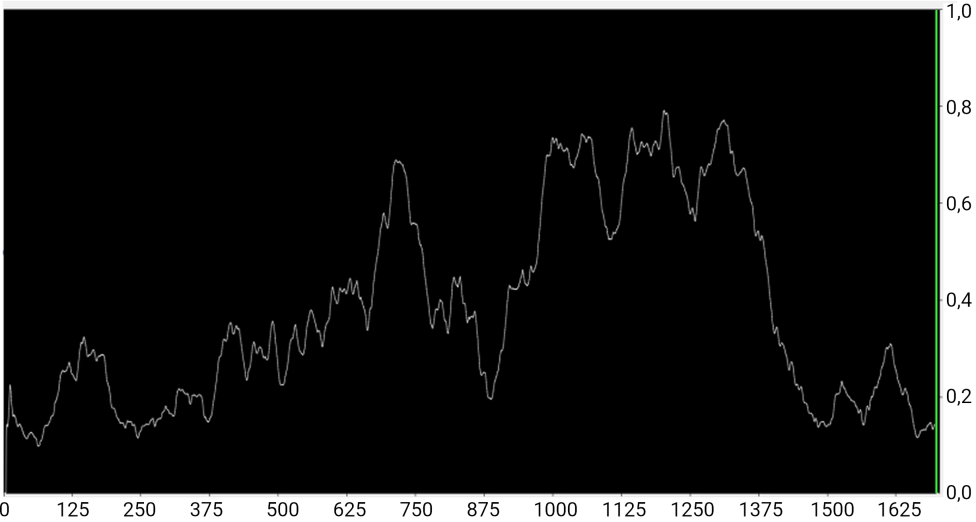


The 5^th^ session: the number of epochs for averaging is 50


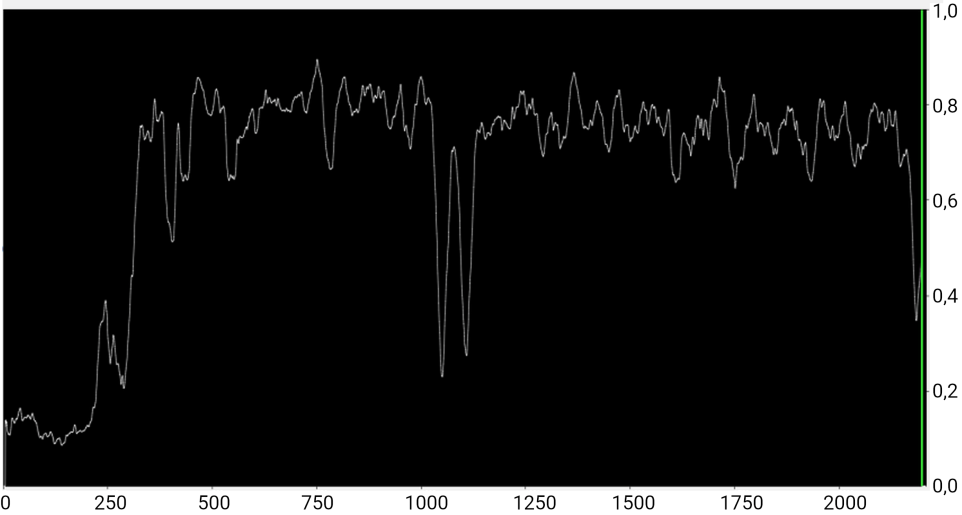


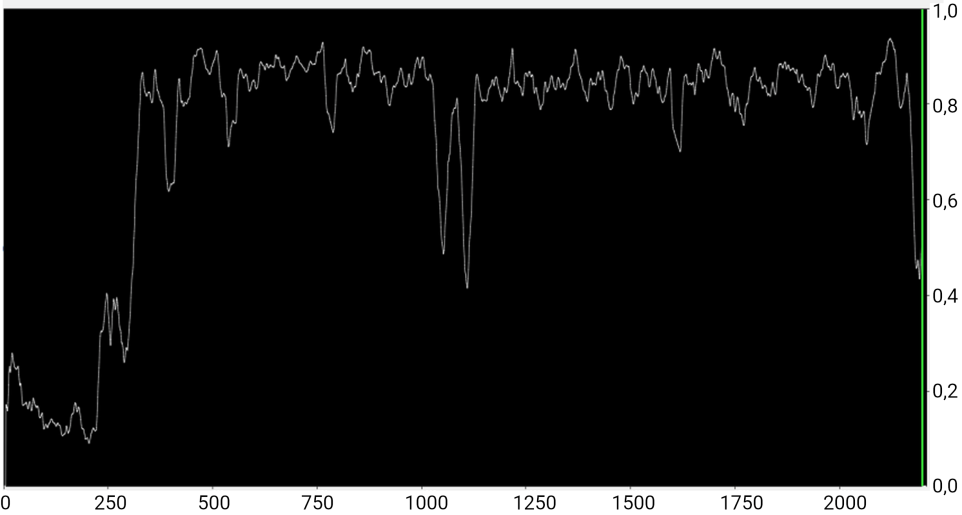


The 6^th^ session: the number of epochs for averaging is 50


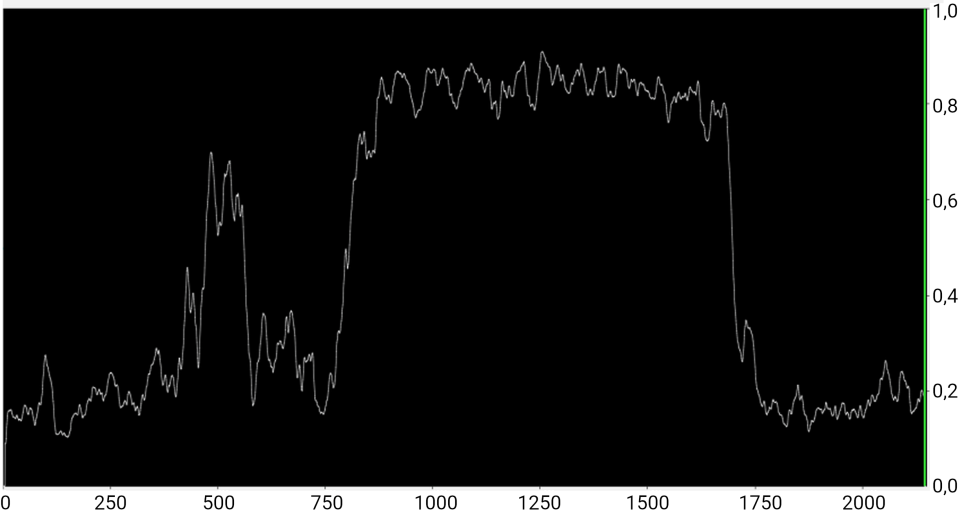


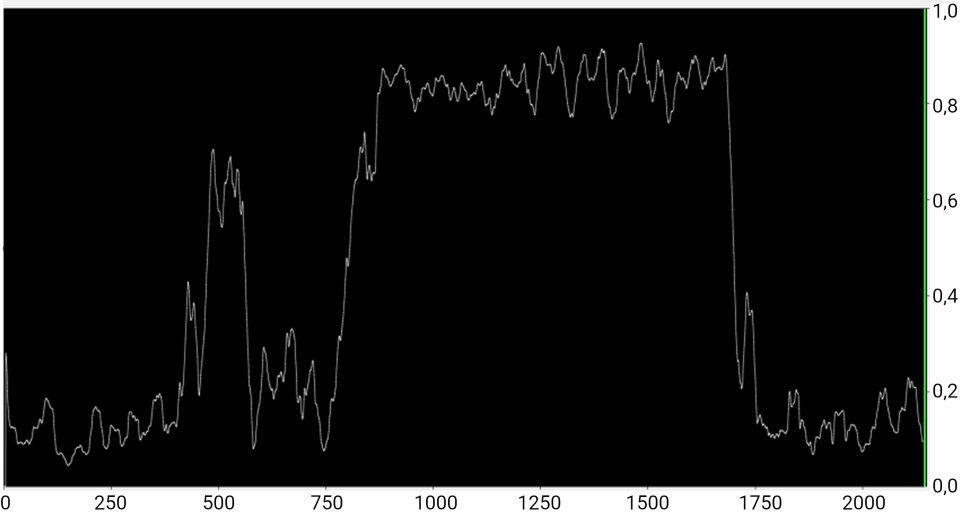


The pair of curves for the 7^th^ session of this patient is displayed in Figures 2 in the main text

**Patient E**, the band used is 4-15 Hz

The 2^nd^ session: the number of epochs for averaging is 150


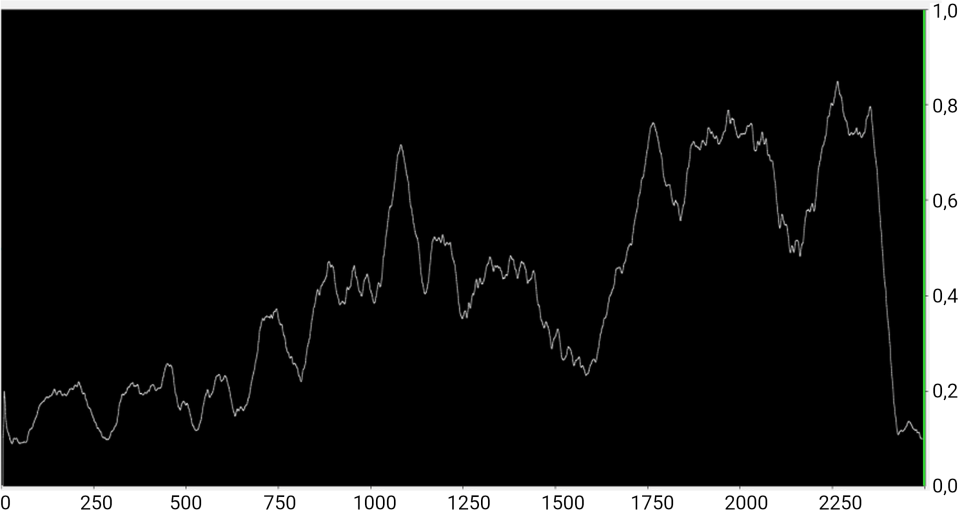


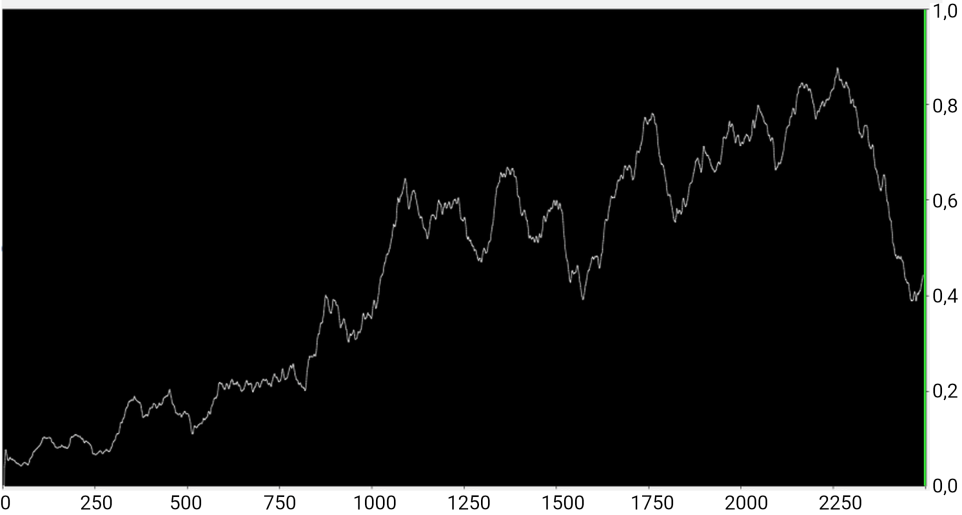


The 3^rd^ session: the number of epochs for averaging is 50


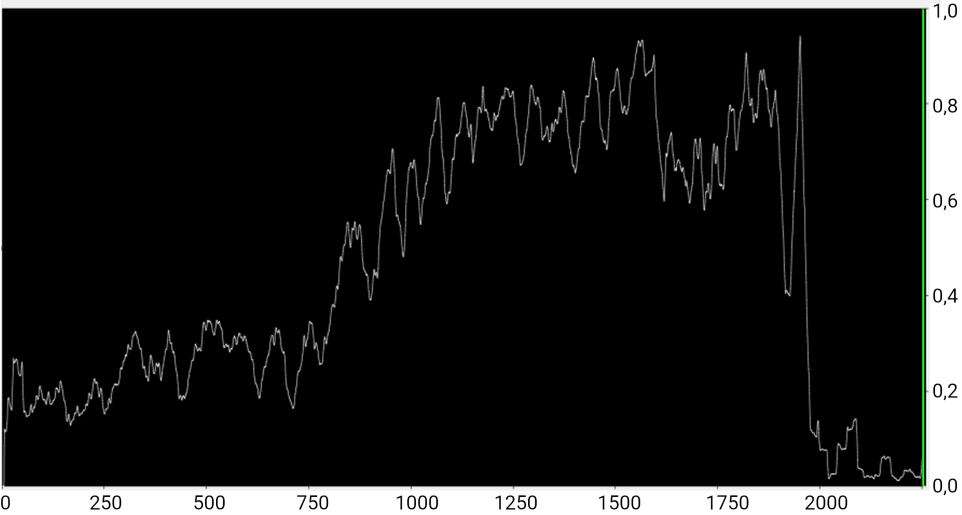


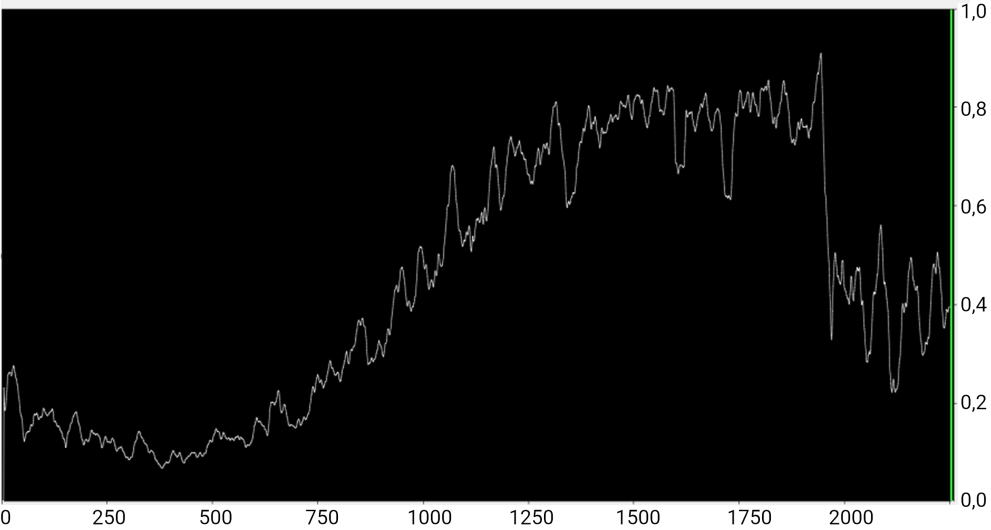


The 4^th^ session: the number of epochs for averaging is 150


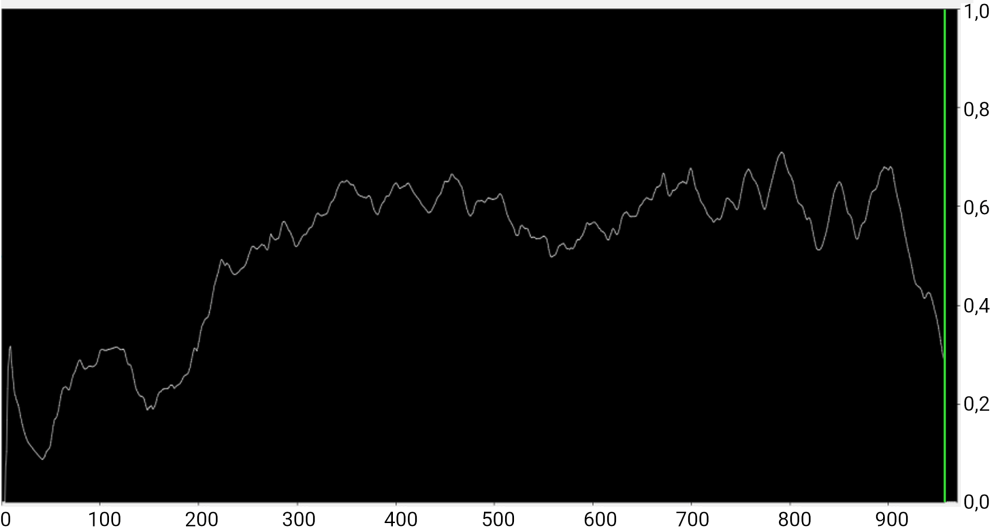


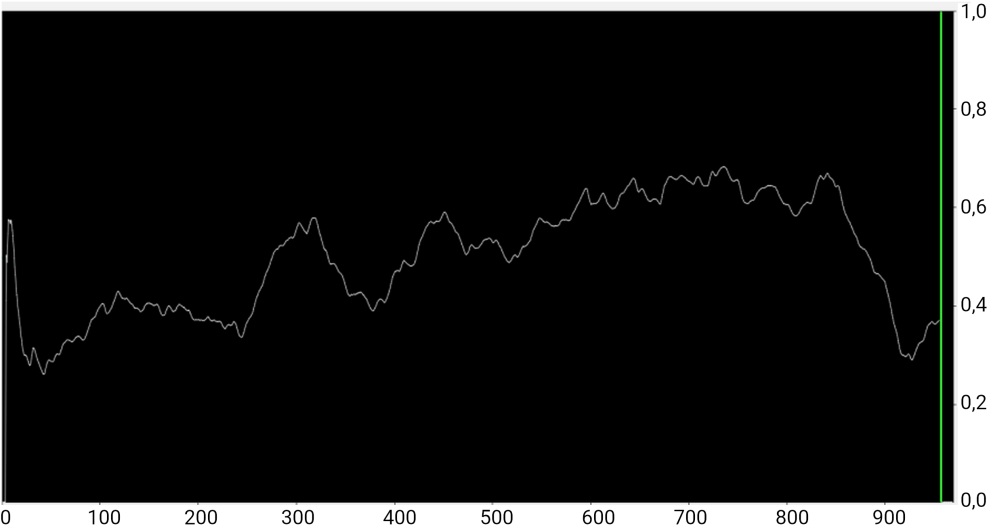


The 5^th^ session was excluded from the analysis

The 6^th^ session: the number of epochs for averaging is 100


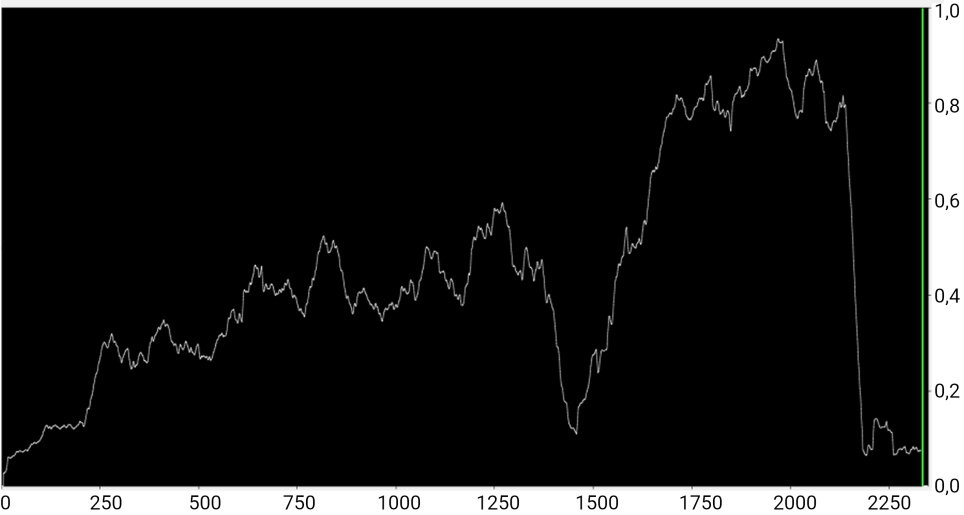


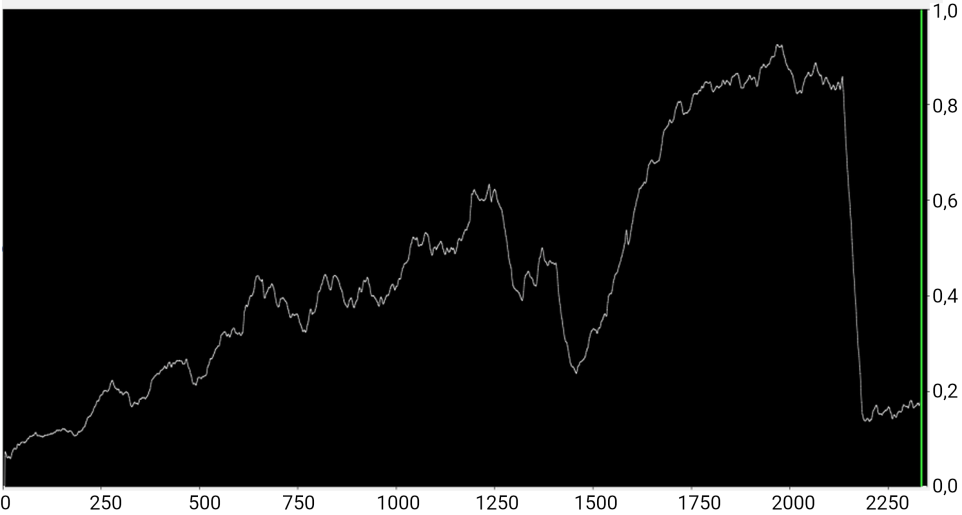


**Patient G**, the band used is 1.5-8 Hz

The 2^nd^ session: the number of epochs for averaging is 100


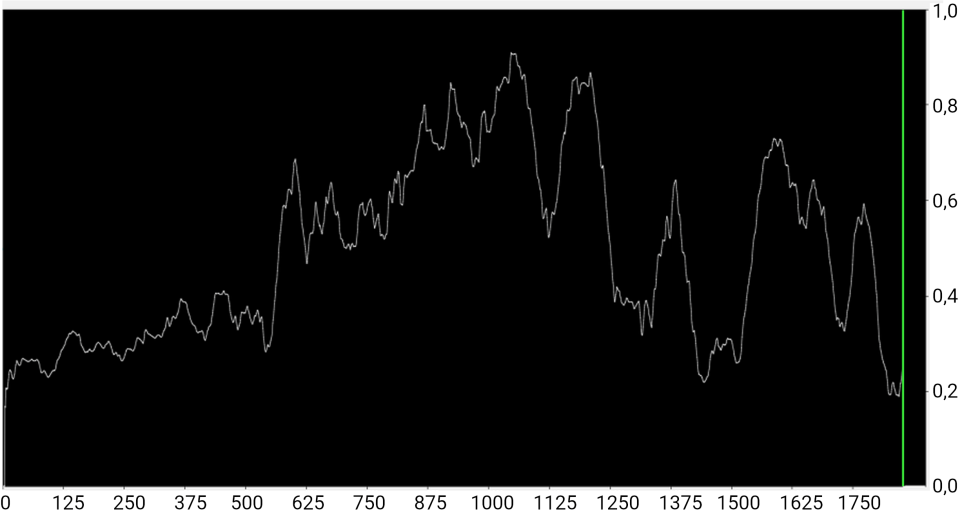


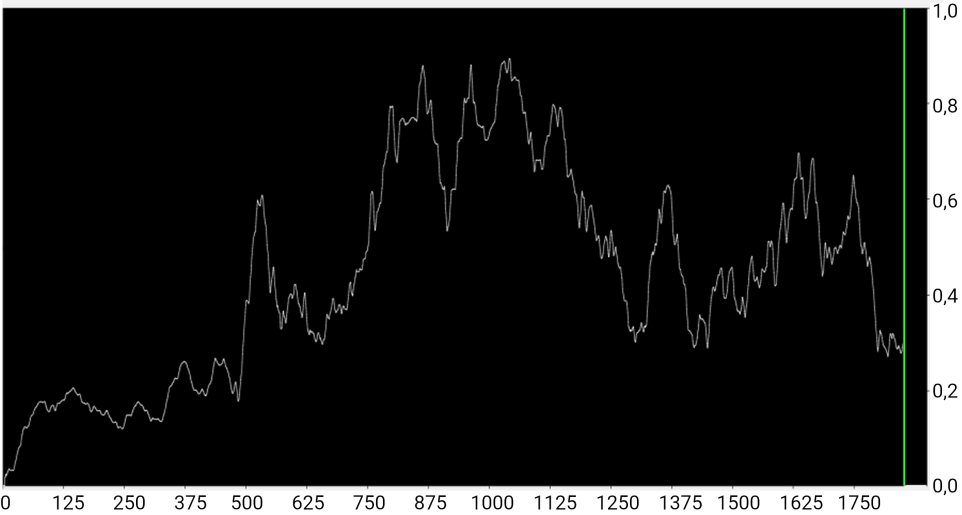


The 3^rd^ session: the number of epochs for averaging is 100


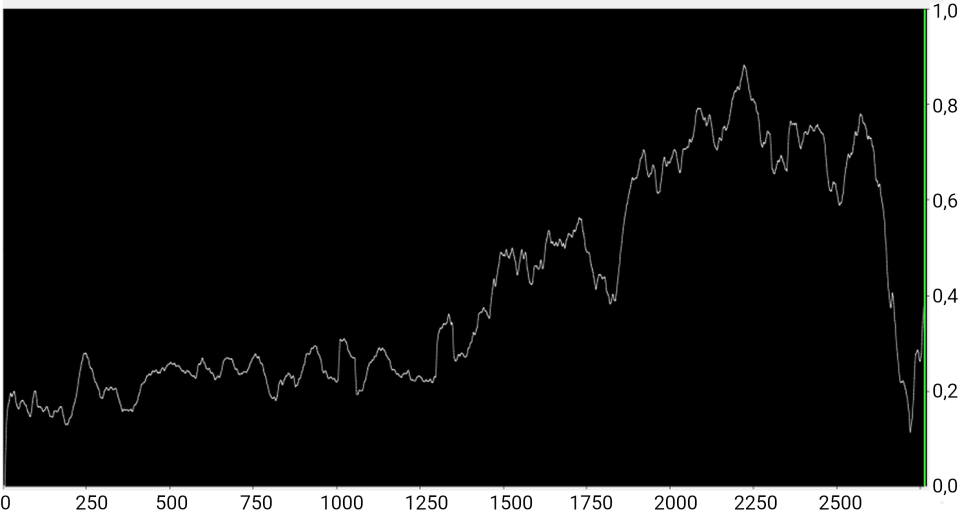


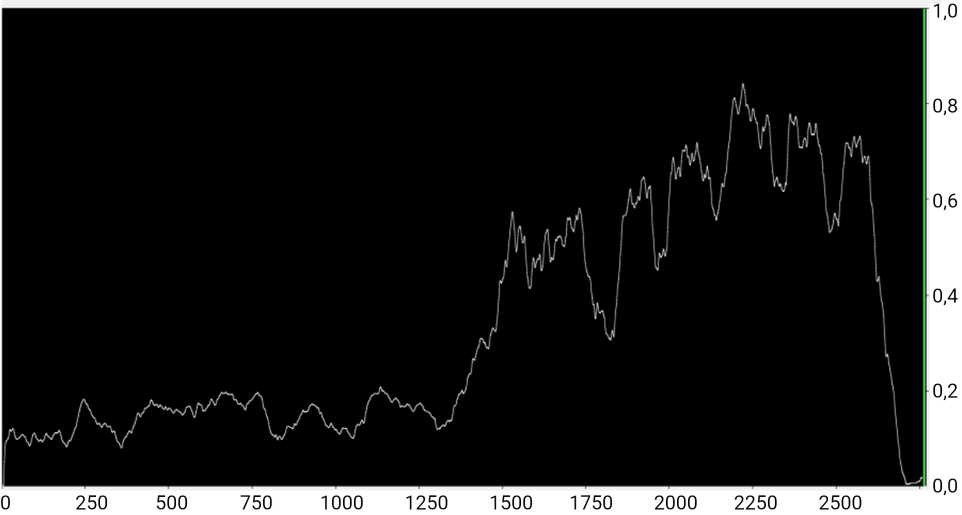


The 4^th^ session: the number of epochs for averaging is 100


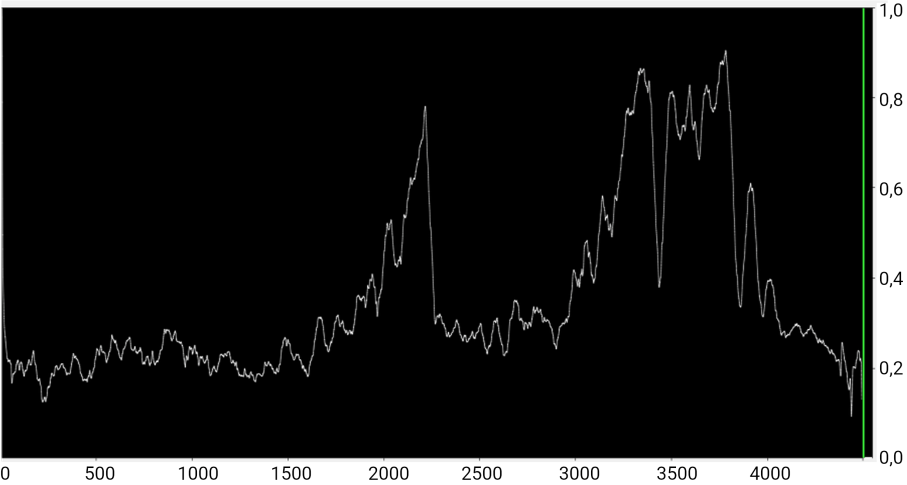


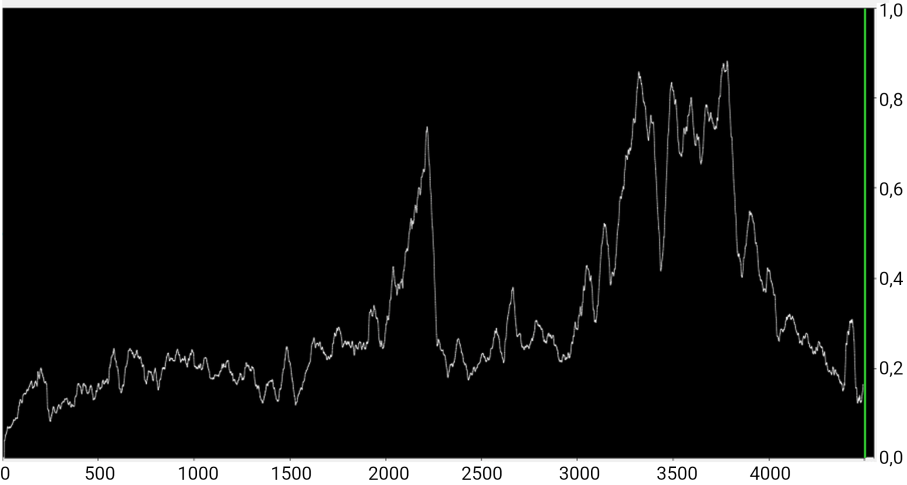


**Patient S**, the band used is 1.5-8 Hz

The 2^nd^ session: the number of epochs for averaging is 100


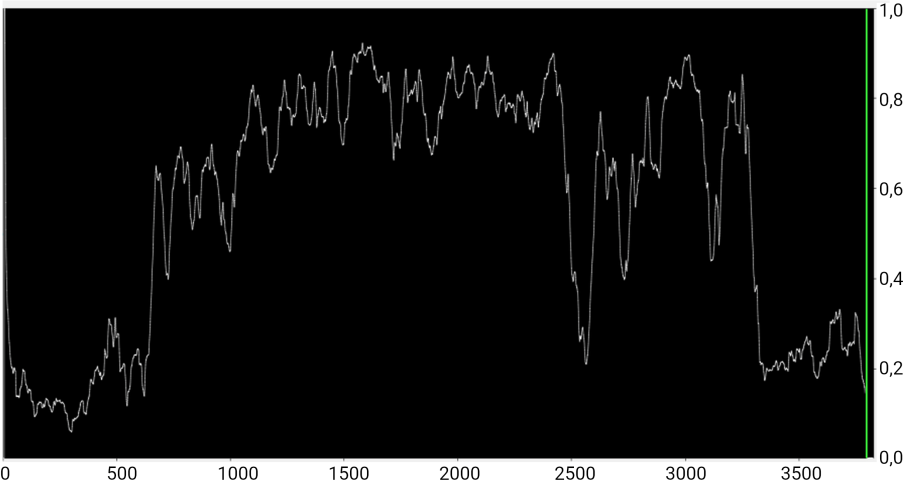


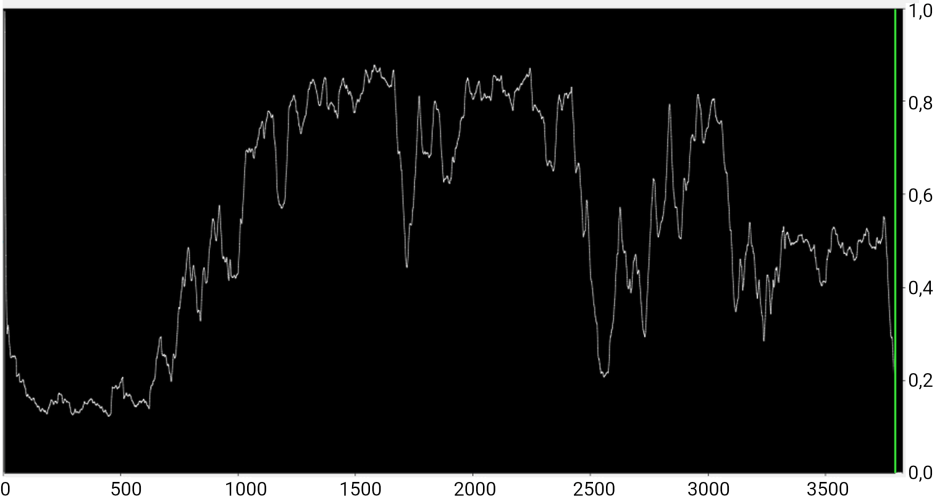


The 3^rd^ session: the number of epochs for averaging is 100


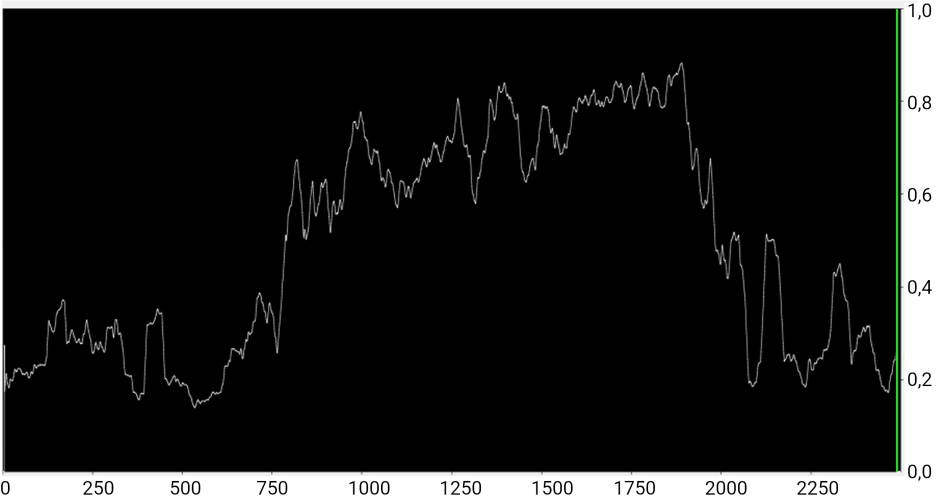


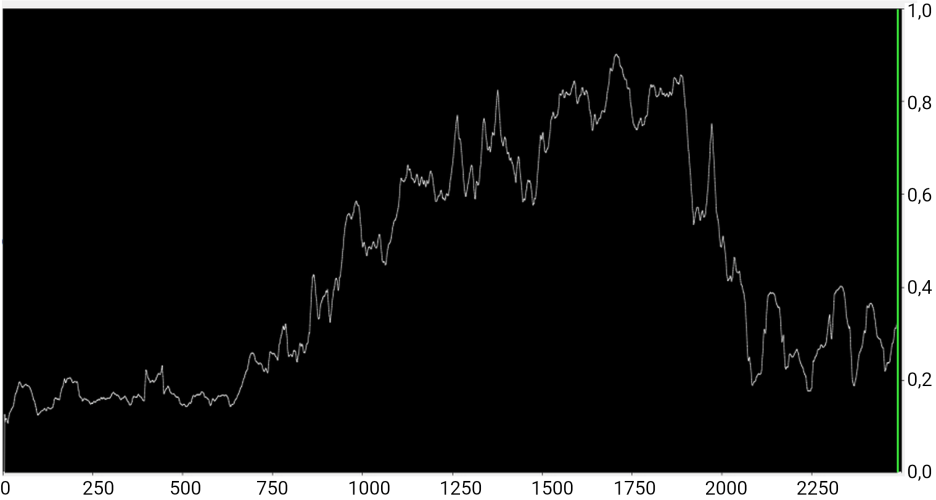


**Patient O**, the band used is 4-15 Hz

The 2^nd^ session: the number of epochs for averaging is 50


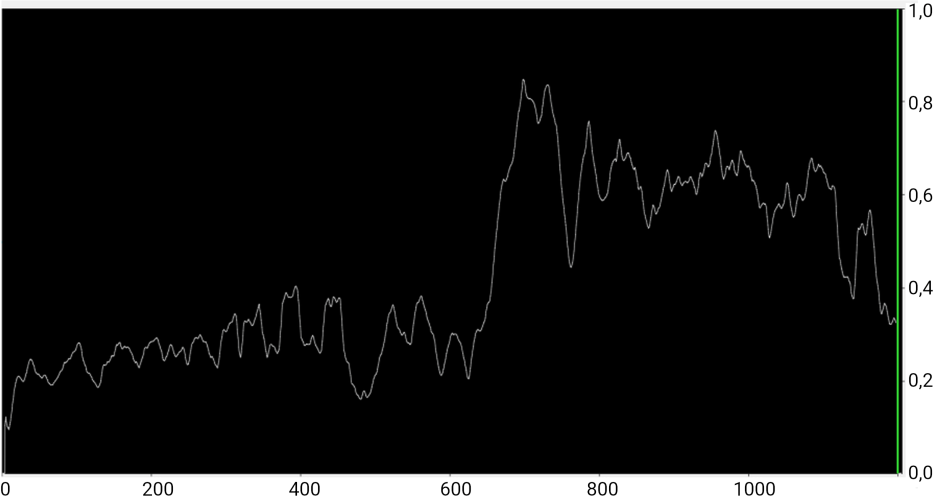


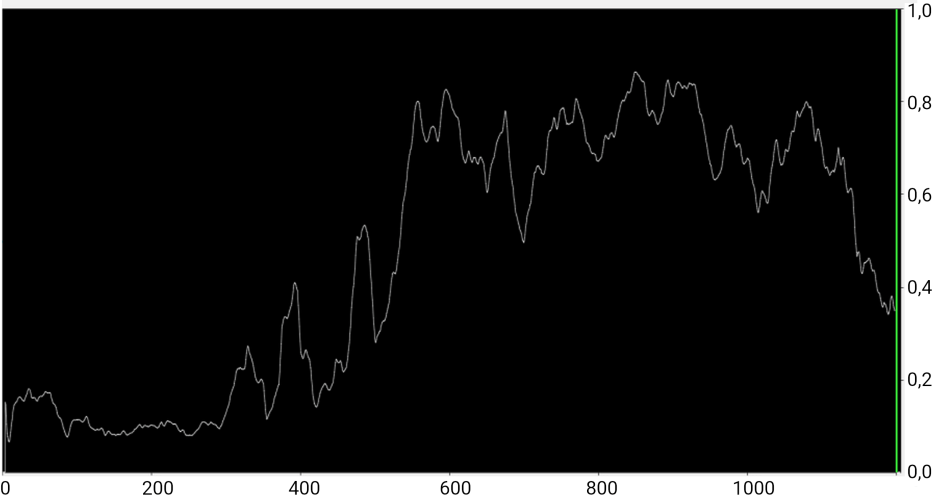


**Patient N**, the band used is 1.5-45 Hz

The 2^nd^ session: the number of epochs for averaging is 150


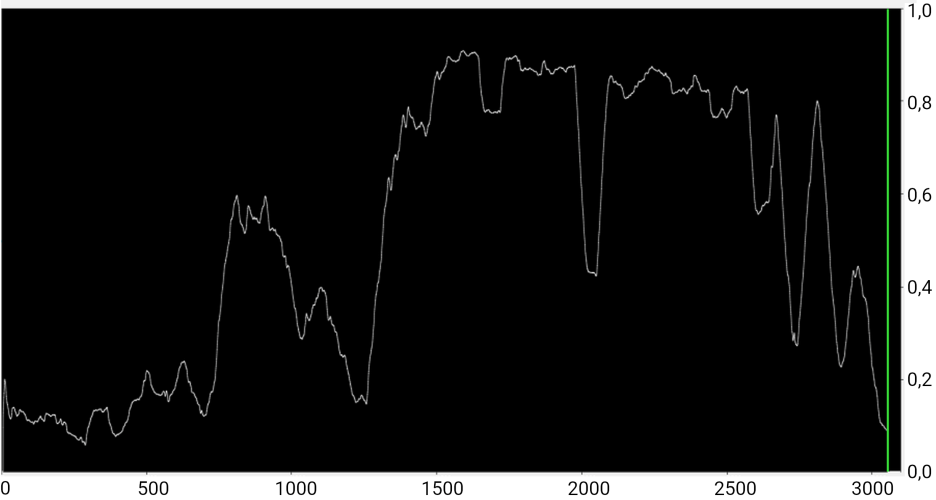


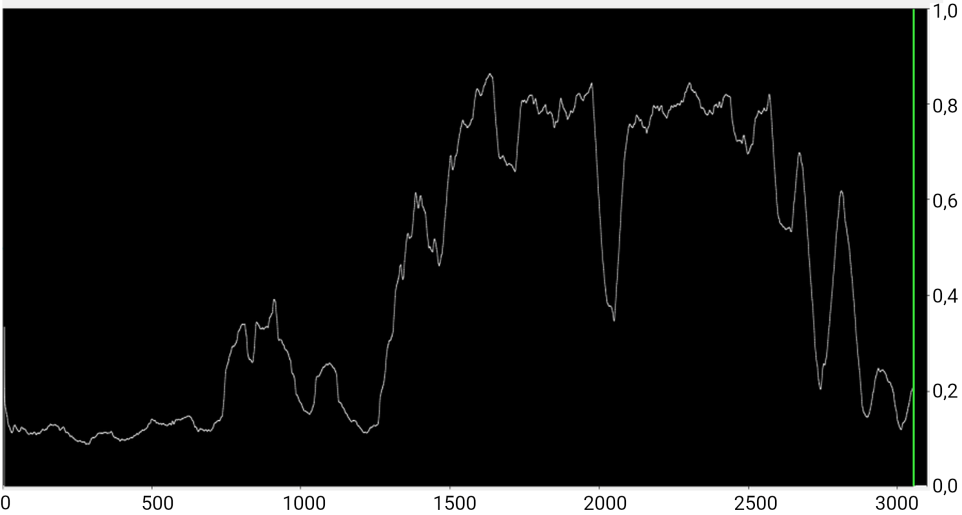


**Patient V**, the band used is 1.5-45 Hz

The 2^nd^ session: the number of epochs for averaging is 100


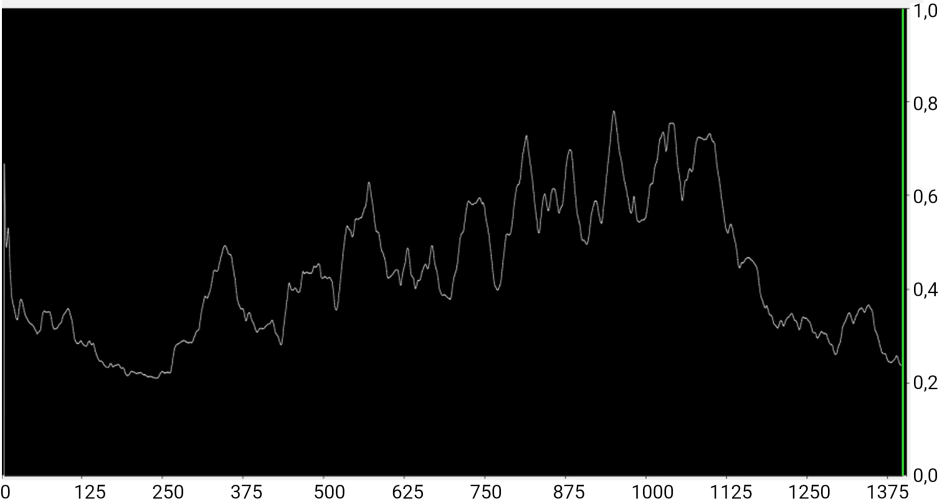


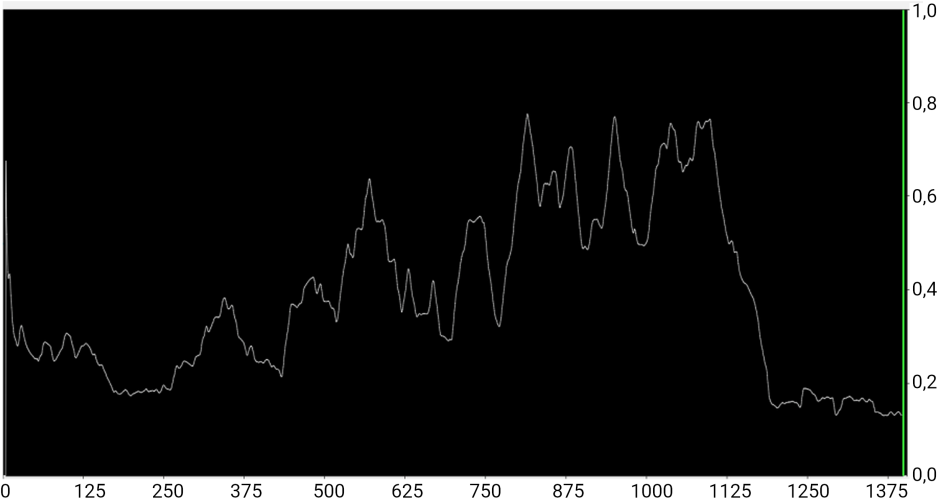


**Patient C**, the band used is 4-15 Hz

The 2^nd^ session: the number of epochs for averaging is 50


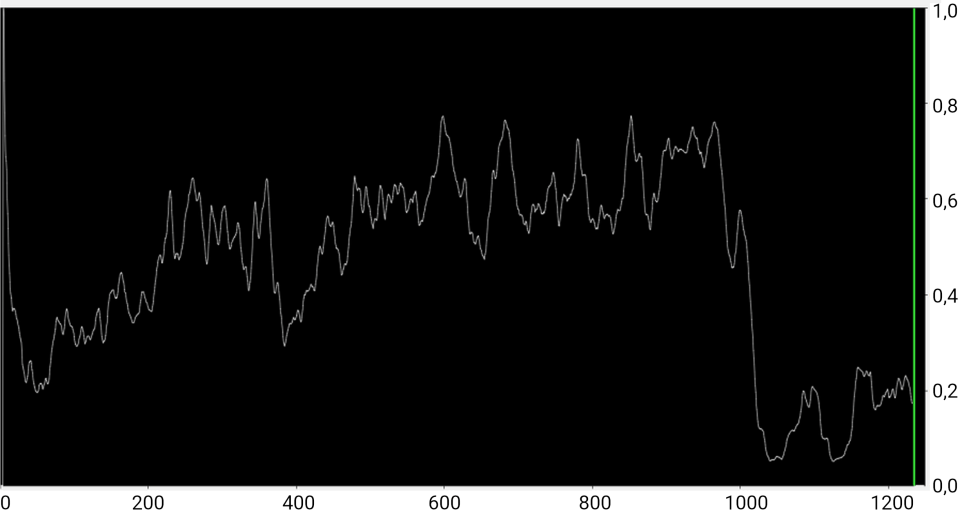


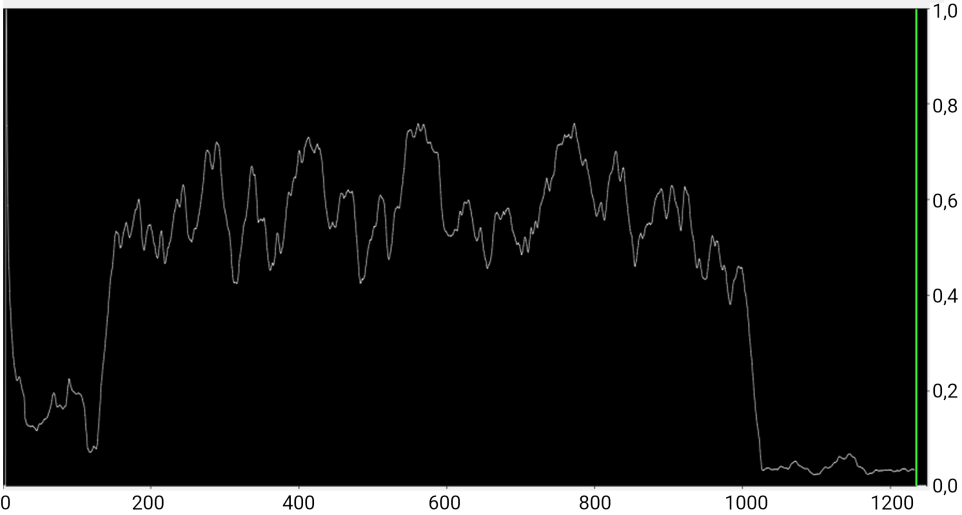


As a result of qualitative analysis based on post-session patients' reports, we found that a Predictive curve was generally able to reflect the phenomenology of a session. To demonstrate this in detail, we could use the example from Figure 2(A) from the main text. There, we can highlight several quite distinct periods in the curve of Patient A. For about the first 200 seconds, the probability values of the deep state were at a low level, which corresponded to calm wakefulness with eyes closed. Then hypnotic induction followed. Since it was the seventh session with this patient and he had already developed the skill to quickly achieve a deep state, the curve rises quite abruptly, but for some time (from about 200th to 500th seconds), it is undulating. This was expressed in the patient's further report that he periodically "plunged deeper", but briefly "surfaced" from time to time and that he remembered this part of the session in fragments. There was a fluctuating character of awareness in this period, according to the description of subjective experience. From about the 500th second, the curve values stabilise around 0.8, with only minor fluctuations, and the patient was completely unaware during this part of the session. Concise therapeutic suggestions had been made, and, since about the 900th second, the awakening phase had started by the reverse counting from "5" to 1," with full awakening on the count of "1" (without opening the eyes to avoid oculographic artefacts). This phase lasted for about 1 minute. However, as seen from the curve, until the moment when the hypnotherapist pronounced the number "1" (959 s), the patient had been in a deep state. Immediately after that, there was a sharp exit, followed by some time of baseline EEG registration with the eyes closed (from 960 to 1105 seconds). The Predictive curves of all the other participants and sessions (the upper plots) generally describe the peculiarities of each session according to the same principle. A relatively accurate reflection of the session phenomenology in the Predictive curve configuration could indirectly indicate that our approach may have high predictive power. This tentative judgement can be underpinned by comparing the graphs of the Predictive and the Native curves of each second and subsequent session of the patients. We can see that their configurations related to a given session generally coincide if a model of high classification accuracy was used to obtain the curves. For example, Figure 2(B) (the Native) shows the periods of hypnotic depth dynamics that are the same as those we were able to observe on the Predictive curve.
