# Supplementary material for: Real-time assessment of hypnotic depth, using an EEG-based brain-computer interface: a preliminary study
Source: BMC Res Notes. 2023 Oct 24;16:288. doi: 10.1186/s13104-023-06553-2 (PMC10599062; doi:10.1186/s13104-023-06553-2)
Supplement: Supplementary file 4 — Supplementary Material 4 (“Supplements D”) [file 13104_2023_6553_MOESM4_ESM.docx]

**Supplements D**

The classification models trained in the first sessions, in general, accurately predicted the actual states of the patients in the next sessions. As expected, the accuracy is lower than with the 10-fold cross-validation, and this reflects the natural variability in electrophysiological characteristics of different sessions. Nevertheless, the classification models from Patients G, N, and V for all four frequency ranges gave very high average accuracy, in some cases above 95%. In Patients A and O, only one of the four bands (1.5-8 Hz for Patient A and 1.5-45 Hz for Patient O) gave unsatisfactory results, while all the others had high predictive power. Two bands gave good results in Patients S (1.5-8 and 1.5-14 Hz) and C (1.5-45 and 4-15 Hz).

The greatest difficulties occurred with Patient E alone, where the classification accuracy was quite high only in the 4–15 Hz band. We assume that the reason for this is that she was the only patient in our study whose experimental conditions were maximally different from session to session. The room and body position were changing: three sessions, including the calibration session, were conducted in a sitting position, and the other three were conducted in a lying position. There were very long intervals between visits (the gap between the first and last sessions was more than a year). Most of the experiments with her were accompanied by artefacts in both the low-frequency (approximately 1.5–3 Hz) and high-frequency (more than 18 Hz) ranges.

The high-frequency artefacts were due to the myogram, which most likely occurred because of the gradually growing uncomfortable position of the head as a result of increasing relaxation of neck muscles. Because of the artefacts, one of the sessions (#5) with this patient was excluded from the analysis. All of this led to the fact that the EEG calibration recording (the first session) gave a classification model that was accurat only in the band free of both low and high frequencies, i.e., in the range from 4 to 15 Hz. As seen from Table 1(b), there was not only high classification accuracy but also very high stability of this accuracy in this band from session to session (SD = 1.9). We detected this band at the beginning of our second and subsequent sessions with her and used it there.

We also assume that the low-frequency artefacts in Patient E could be due to such a common phenomenon as drift. The Predictive curves of her second (on 1.5–45 Hz) and third (on 1.5–45 and 1.5–14 Hz) sessions had a very similar shape to the Native curves, but with the only difference that they were "raised" above the zero value, and it was possible to increase the classification accuracy of these prediction models by subtracting a certain constant value from the feature vector in Scenario-IV (see the notes below Table 1). The lack of classification accuracy in the 1.5–8 and 1.5–14 Hz ranges in Patient C was also associated with low-frequency artefacts.

Consequently, for the BCI to be used for hypnosis, we could recommend creating conditions with a minimised artefact probability, i.e., conducting sessions in the same room, body position, etc. We could also recommend pre-training several models based on different frequency bands while calibrating, as was done in our study. If the artefacts are unavoidable and the initially applied model at the very beginning of the second and subsequent session demonstrates incorrect waking state prediction, then it is necessary to replace it immediately with another model that is trained on a narrower frequency band, with the exclusion of the slow waves (which may be associated with drift) or high-frequency activity (related to the myogram). Another way to cope with this problem is to correct the scenario by subtracting an empirically selected constant value from the feature vector, thus eliminating the drift.
